# Oxygen availability strongly affects chronological lifespan and thermotolerance in batch cultures of *Saccharomyces cerevisiae*

**DOI:** 10.15698/mic2015.11.238

**Published:** 2015-10-21

**Authors:** Markus M. Bisschops, Tim Vos, Rubén Martínez-Moreno, Pilar T. Cortés, Jack T. Pronk, Pascale Daran-Lapujade

**Affiliations:** 1 Department of Biotechnology, Delft University of Technology, Delft, The Netherlands.; 2 Instituto de Ciencias de la Vid y del Vino, CSIC, Universidad de La Rioja, Gobierno de La Rioja, Logroño, Spain.; 3 Current address: Division of Systems and Synthetic Biology, Department of Biology and Biological Engineering & The Novo Nordisk Foundation Center for Biosustainability, Chalmers University of Technology, Gothenburg, Sweden.; 4 Current address: Quercus Europe S.L., L’Hospitalet de Llobregat, Catalonia, Spain.

**Keywords:** chronological lifespan, thermotolerance, stationary phase, anaerobiosis, energetics, transcriptional response, conditioning

## Abstract

Stationary-phase (SP) batch cultures of *Saccharomyces cerevisiae*, in which growth has been arrested by carbon-source depletion, are widely applied to study chronological lifespan, quiescence and SP-associated robustness. Based on this type of experiments, typically performed under aerobic conditions, several roles of oxygen in aging have been proposed. However, SP in anaerobic yeast cultures has not been investigated in detail. Here, we use the unique capability of *S. cerevisiae* to grow in the complete absence of oxygen to directly compare SP in aerobic and anaerobic bioreactor cultures. This comparison revealed strong positive effects of oxygen availability on adenylate energy charge, longevity and thermotolerance during SP. A low thermotolerance of anaerobic batch cultures was already evident during the exponential growth phase and, in contrast to the situation in aerobic cultures, was not substantially increased during transition into SP. A combination of physiological and transcriptome analysis showed that the slow post-diauxic growth phase on ethanol, which precedes SP in aerobic, but not in anaerobic cultures, endowed cells with the time and resources needed for inducing longevity and thermotolerance. When combined with literature data on acquisition of longevity and thermotolerance in retentostat cultures, the present study indicates that the fast transition from glucose excess to SP in anaerobic cultures precludes acquisition of longevity and thermotolerance. Moreover, this study demonstrates the importance of a preceding, calorie-restricted conditioning phase in the acquisition of longevity and stress tolerance in SP yeast cultures, irrespective of oxygen availability.

## INTRODUCTION

Just like other living organisms, *Saccharomyces cerevisiae *cells age and have a finite chronological lifespan. The similarity of cellular processes in *S. cerevisiae *to those in higher eukaryotes and its accessibility to a wide range of experimental techniques have made this yeast a popular model for studying chronological aging of metazoan cells 1,2,3,4. Chronological aging of *S. cerevisiae* is typically studied in aerobic batch cultures, in which growth arrest and quiescence are triggered by exhaustion of the available carbon sources in the growth medium 5,6. Survival of individual yeast cells in such non-growing, stationary-phase (SP) cultures is then taken as a measure for their chronological lifespan (CLS). Over the past decade, studies on SP yeast cultures have contributed to our understanding of cellular mechanisms involved in aging, and several underlying cellular mechanisms were also found in higher eukaryotes 7.

Calorie restriction has been shown to extend lifespan in organisms ranging from yeast to man, with studies on many organisms pointing at an important role of nutrient-signaling cascades 8. Turn-over of damaged macromolecules, and in particular proteins, has similarly been identified as a key process in aging in many organisms 9. A third universal factor implicated in aging is respiration and, in particular, the associated formation of reactive oxygen species (ROS), which has been shown to enhance aging-related cellular deterioration in many organisms 10. However, ROS have also been implicated in beneficial effects. In particular, mild ROS stress has been proposed to contribute to CLS extension by inducing stress-resistance genes, a phenomenon known as hormesis 11,12. Similarly, increased mitochondrial respiration and ROS production rates in calorie-restricted yeast cultures have been linked to CLS extension 13,14,15.

ROS generation is not necessarily the only mechanism by which respiration and oxygen can affect CLS. In aerobic, glucose-grown batch cultures of *S. cerevisiae*, a fast and predominantly fermentative growth phase on glucose is followed by a second, respiratory growth phase in which the fermentation products ethanol and acetate are consumed 16. This second growth phase, known as post-diauxic phase, is characterized by slow growth. During the post-diauxic phase, genes involved in SP are already expressed at an elevated level, as well as some features associated with SP cultures, such as increased stress resistance 6. In anaerobic cultures of *S. cerevisiae*, the absence of oxygen prevents a respiratory post-diauxic growth phase. Instead, a phase of fast, fermentative exponential growth on glucose is immediately followed by SP, in which maintenance of viability and cellular integrity depends on metabolism of storage compounds. *S. cerevisiae* cells can contain two types of storage polymers: the storage carbohydrates trehalose and glycogen, and fatty acids, which are mostly stored in the form of di- and triacylglycerol esters 17,18,19. In the absence of oxygen, yeast cells cannot catabolize fatty acids by β-oxidation and, moreover, conversion of storage carbohydrates via alcoholic fermentation yields 5-8 fold less ATP than their respiratory dissimilation 20.

Previous studies on the role of respiration in aging were predominantly based on the use of respiration-deficient *S. cerevisiae *mutants (e.g. ρ^0^ strains and other mutants) 21,22,23,24,25 and respiratory inhibitors 14. These approaches, however, have several drawbacks. Firstly, mitochondria are not only involved in respiration, but also in essential anabolic reactions (e.g., assembly of iron-sulfur complexes, amino acid biosynthesis and long-chain lipid biosynthesis 26). Studies on petite or ρ^0^ mutants may therefore cause unwanted ‘side-effects’ resulting from the absence or inefficiency of mitochondrial processes, rather than from direct effects of oxygen or respiration on aging. For example, the absence of mitochondrial DNA influences crosstalk between these organelles and the nucleus 27. Furthermore, inhibition of respiration may result in reduced ROS levels 14, but can also result in ROS accumulation 28, depending on the intervention chosen. In addition, ROS may still be produced by other oxygen-consuming processes in yeast, such as disulfide-bond formation during oxidative protein folding 29.

Surprisingly, while *S. cerevisiae* is unique among yeasts and eukaryotes for its ability to grow fast under fully aerobic as well as strictly anaerobic conditions 30, this ability has not been used to systematically investigate the impact of oxygen availability on entry into SP, on longevity and on robustness. The goal of the present study was therefore to investigate the impact of oxygen availability on yeast physiology in SP cultures. More specifically, we investigated whether the post-diauxic phase and respiratory mobilization of storage compounds in aerobic cultures affects CLS and thermotolerance during SP. To this end, aerobic and anaerobic bioreactor batch cultures of *S. cerevisiae *were grown into SP and subjected to detailed physiological and transcriptome analyses.

## RESULTS

### Anaerobicity reduces chronological lifespan and stress resistance in stationary phase cultures

**Figure 1:**
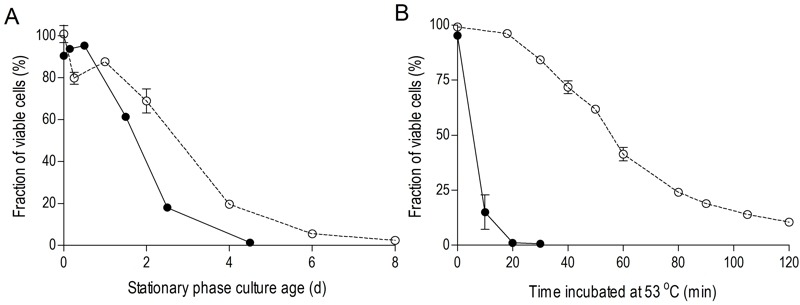
FIGURE 1: Chronological life span and thermotolerance of stationary-phase cultures is much lower under anaerobic than under aerobic conditions. Chronological life span **(A)**: survival expressed as ratio of colony forming units divided by the number of cells plated, during aerobic and anaerobic SP cultures. Time point zero indicates the onset of SP, which corresponds to exogenous glucose exhaustion in anaerobic cultures and exhaustion of all exogenous carbon sources including ethanol and organic acids in aerobic cultures. Thermotolerance **(B)**: loss of viability after sudden exposure of cells from aerobic and anaerobic cultures during early SP to 53°C. Open symbols (○) represent aerobic cultures, closed symbols (●) anaerobic cultures. Data represent the average and SEM of measurements on independent duplicate cultures.

To investigate the impact of oxygen availability on chronological lifespan in SP cultures of *S. cerevisiae, *survival kinetics were analyzed during SP in aerobic and anaerobic, glucose-grown bioreactor cultures. In aerobic cultures, the percentage of cells capable of colony formation on complex-medium agar plates typically decreased to ca. 2% in the 8 days following onset of SP, i.e. after exhaustion of all exogenous carbon sources including ethanol and organic acids (Figure 1A). Viability of anaerobic cultures decreased much faster, reaching values below 1% within 4.5 days after the onset of SP, that is after all exogenous glucose was consumed (Figure 1A).

Increased thermotolerance is a well-documented characteristic of SP cultures of *S. cerevisiae* 31,32. Indeed, half of the cells in samples from aerobic, early-SP cultures survived a 60-min exposure to 53°C. Notably, up to 20 minutes incubation at 53°C hardly affected viability, suggesting that cells were well capable of repairing heat-induced damage during this period. In contrast, fewer than 20% of the cells from anaerobic early-SP cultures survived a 10-min incubation at this temperature (Figure 1B). Implementation of anaerobic conditions during sampling and heat-shock assays did not significantly affect this difference, indicating that heat-induced loss of viability was not influenced by exposure of anaerobically grown cells to oxygen during the assays (data not shown). Furthermore, washing of cells prior to the heat-shock experiments did not influence heat-shock resistance, indicating that the presence of low (< 1 g/L) ethanol concentrations in the assays did not cause the low thermotolerance of cells from anaerobic SP cultures.

### Oxygen availability strongly affects the transcriptome of SP cultures

In aerobic yeast cultures, entry into SP is accompanied by a range of physiological changes that enhance survival in harsh, nutrient-poor environments 31. This adaptation coincides with a vast transcriptional reprogramming 33,34,35 that includes up-regulation of genes involved in resistance mechanisms to a wide array of stresses. Currently, no transcriptome data are available in the literature on anaerobic SP cultures of *S. cerevisiae*.

A transcriptome analysis, performed on culture samples taken 4 h after the onset of SP, revealed that a quarter of the yeast genome (1452 genes, Supplemental Table S1) was differentially expressed (fold-change cut-off of 2.0 and adjusted P-value below 0.05) in aerobic and anaerobic SP cultures. Among these genes were several genes known to be regulated by the heme and oxygen dependent transcription factors Hap1 and Rox1 36,37,38. Approximately 40% of the differentially expressed genes (574 genes, Supplemental Table S1) were transcribed at higher levels in the aerobic SP cultures. This gene set showed a strong overrepresentation of genes involved in fatty acid metabolism and, in particular, in β-oxidation (Table 1). This set of genes was also strongly enriched for genes that were up-regulated during SP in previous studies performed in shake flasks 34 (Table 1). Examples included the SP-genes *SPG1*, *SPG3*, *SPG4*, *SPG5*, and *SSA3*, which encodes a stress-induced ATPase. Furthermore, a significant number of genes (54) induced by the environmental stress response 39 was expressed at higher levels in aerobic SP cultures than in their anaerobic counterparts (Table 1).

**Table 1:** Functional categories overrepresented among genes with different expression levels in aerobic and anaerobic stationary phase cultures. ^a^Bonferroni-corrected P-value cut-off of 0.05 was used and P-values indicate the probability of finding the same numbers of genes in a random set of genes. Functional categories are obtained from the Gene Ontology set or, in italic font, directly from literature references. Details can be found in supplemental table S2.

**Category description**	**# of genes in dataset**	**# of genes in category**	**P-value^a^**
**574 genes with higher expression in aerobic stationary phase cultures**			
*Genes induced in stationary phase 34*	53	122	1.2 ^.^ 10^-22^
*Genes induced by environmental stress response 39*	54	281	3.2 ^.^ 10^-6^
Fatty acid metabolic process	13	29	5.4 ^.^ 10^-4^
Fatty acid beta-oxidation	7	9	2.0 ^.^ 10^-3^
Transmembrane transport	52	303	3.8 ^.^ 10^-3^
Glyoxylate cycle	6	8	1.7 ^.^ 10^-2^
**878 genes with higher expression in anaerobic stationary phase cultures**			
Translation	131	345	4.1 ^.^ 10^-28^
*Genes induced by environmental stress response 39*	78	281	1.7 ^.^ 10^-8^
Mitochondrial translation	36	81	1.9 ^.^ 10^-8^
Oxidation reduction	74	270	1.6 ^.^ 10^-6^
Metabolic process	93	389	2.6 ^.^ 10^-5^
Response to stress	49	161	3.2 ^.^ 10^-5^
Heme biosynthetic process	10	12	1.6 ^.^ 10^-4^
Methionine metabolic process	11	15	3.5 ^.^ 10^-4^
Sulfate assimilation	9	11	1.0 ^.^ 10^-3^
Porphyrin biosynthetic process	8	9	1.4 ^.^ 10^-3^
Carbohydrate metabolic process	28	93	4.2 ^.^ 10^-2^
Glycolysis	14	32	4.6 ^.^ 10^-2^
Methionine biosynthetic process	14	32	4.6 ^.^ 10^-2^

Most of the genes that were differentially expressed in aerobic and anaerobic SP cultures (60%, 878 genes, Supplemental Table S1) showed a higher transcript level under anaerobic conditions. This gene set showed an overrepresentation of genes that were previously shown to be expressed at high levels in anaerobic yeast cultures and which were therefore not necessarily related to SP. This subgroup included genes involved in heme synthesis 38 and members of the multi-gene seripauperin family 40 (Table 1 and Supplemental Table S2). Interestingly, a strong overrepresentation (131 out of 345 genes) was found for the GO category ‘translation’ (Table 1). This subset included many genes encoding cytosolic and mitochondrial ribosomal proteins (65 and 24 genes respectively). Furthermore, several genes involved in carbohydrate metabolism, including glycogen metabolism, were expressed at higher levels in anaerobic SP cultures (Table 1). Finally, the set of 878 genes with higher expression in anaerobic SP cultures showed a very strong overrepresentation of genes induced by the environmental stress response 39.

### Anaerobicity negatively affects the energy status of SP cultures

Use of oxygen as an electron acceptor for respiration enables oxidative phosphorylation. As a consequence, ATP yields on glucose, glycogen and trehalose in respiratory cultures can be up to ca. 8-fold higher than in anaerobic, fermentative cultures 20,41. Furthermore, since anaerobic yeast cultures cannot catabolize fatty acids, their use as energy reserves is restricted to aerobic cultures. To investigate the impact of storage metabolism and energy status on the short CLS of anaerobic SP cultures, cellular contents of storage materials and adenylate energy charge (a measure for the energetic status of living cells 42) were analysed in aerobic and anaerobic SP cultures.

**Figure 2:**
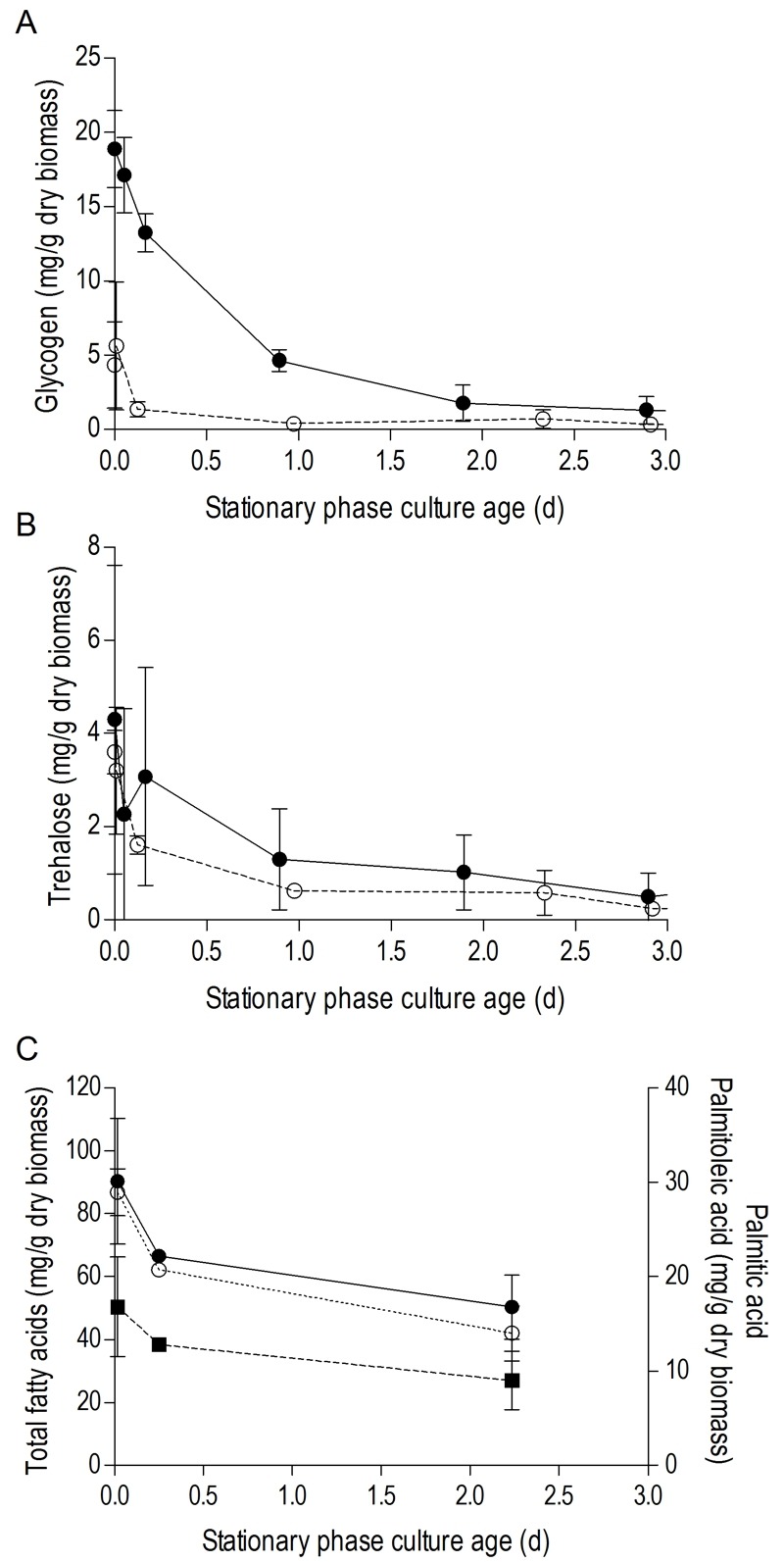
FIGURE 2: Utilization of carbohydrate and lipid storage compounds in aerobic and anaerobic stationary-phase cultures. Cellular contents of glycogen **(A)**) and trehalose **(B)** are shown for the SP of glucose-grown batch cultures of *S. cerevisiae*, grown under aerobic (open symbols, ○) or anaerobic (closed symbols, ●) conditions. Panel **(C)** shows cellular contents of total fatty acids (closed circles, ●), palmitoleic acid (open circles, ○) and palmitic acid (closed squares, ■) in aerobic SP cultures. Time point zero indicates the onset of SP, which corresponds to exogenous glucose exhaustion in anaerobic cultures and exhaustion of all exogenous carbon sources including ethanol and organic acids in aerobic cultures. Average and SD or SEM are shown, calculated from either quadruplicate cultures (glycogen and trehalose) or duplicate cultures (fatty acids), respectively.

**Figure 3:**
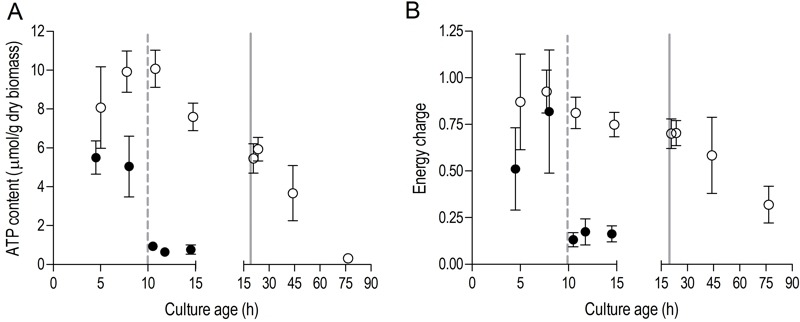
FIGURE 3: Intracellular ATP concentration and adenylate energy charge in aerobic and anaerobic stationary-phase cultures. Cellular ATP content **(A)** and adenylate energy charge **(B)** in aerobic (open symbols, ○) and anaerobic (closed symbols, ●) cultures. The dashed vertical line represents glucose exhaustion and the onset of anaerobic SP, the solid vertical line represents ethanol exhaustion and the onset of aerobic SP. Values are shown as averages of duplicate cultures (+/- SEM).

In aerobic cultures, intracellular pools of trehalose and glycogen were depleted within 1 day after entry into SP (Figure 2A and 2B). Cellular contents of the fatty acids palmitic and palmitoleic acid, also decreased during aerobic SP, but at a much slower rate than the storage carbohydrates and approached 6% of the total fatty acids content. This level is close to the membrane-associated fatty acid content previously reported in *S. cerevisiae* 43, indicating that yeast cells had exhausted most of their reserve lipids after 2 days in SP (Figure 2C). Together with the increased expression of genes involved in β-oxidation in aerobic SP cultures (Table 1), this observation indicates that aerobic SP cells use part of their fatty acids as an endogenous carbon and energy source. In aerobic cultures, the adenylate energy charge was 0.70 (± 0.08) and the intracellular ATP concentration was 5.45 (± 0.76) mM at the onset of SP. These results are in good agreement with published data 44,45. In the days after the onset of SP, both parameters gradually decreased. Two full days after the onset of SP, the adenylate energy charge was still above 0.25 (Figure 3).

Trehalose and glycogen are the only known carbon and energy reserves in anaerobic *S. cerevisiae *cultures. The initial trehalose content and utilization profile in anaerobic SP cultures strongly resembled those observed in aerobic cultures. The initial glycogen concentration in anaerobic SP cultures was ca. four-fold higher than in aerobic SP cultures. Nevertheless, intracellular glycogen was exhausted after 2 days in SP (Figure 2A and 2B). At the onset of SP, intracellular ATP concentration and adenylate energy charge of anaerobic SP cultures were already lower than in aerobic cultures. Moreover, they decreased to very low levels within half a day after the onset of SP (Figure 3).

### Different entry trajectories into SP in aerobic and anaerobic cultures 

**Figure 4:**
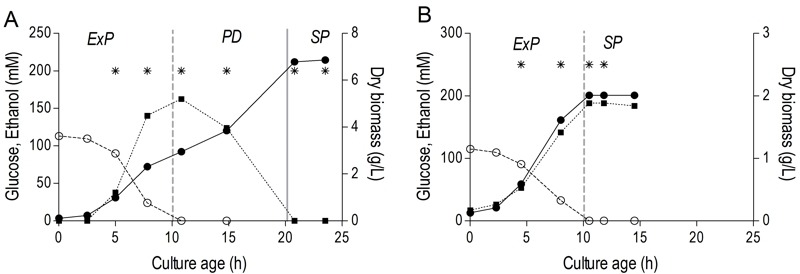
FIGURE 4: Growth phases in aerobic and anaerobic batch cultures. Biomass (closed circles, ●), glucose (open circles, ○) and ethanol (closed squares, ■) concentration during the different growth phases of aerobic **(A)** and anaerobic **(B)** batch cultures of *S. cerevisiae*. The initial phase of exponential growth on glucose (ExP), the following post-diauxic phase of slower growth on non-fermentable carbon sources (PD) and final stationary phase (SP) are indicated. Values shown are from single representative batch cultures, independent replicate cultures yielded essentially the same results. Vertical lines indicate depletion of glucose (dashed line) and of fermentation products (solid line). Asterisks (*) indicate time points at which samples were taken for transcriptome analysis.

The results described above reveal clear differences in transcriptome, energy status, thermotolerance and CLS of aerobic and anaerobic SP cultures. Some of these parameters already differed at the onset of SP, indicating the importance of different ‘entry trajectories’ of aerobic and anaerobic cultures into SP. A major difference between aerobic and anaerobic batch cultures is the absence, in the latter, of a respiratory post-diauxic phase, in which ethanol and minor fermentation products acetate and glycerol are consumed. Growth in the post-diauxic phase, in which metabolism is completely respiratory, is slower than during the preceding glucose phase 46. In this study, the maximum specific growth rate of anaerobic cultures (0.31 ± 0.01 h^-1^) during the glucose phase was lower than that of aerobic cultures (0.39 ± 0.02 h^-1^, Figure 4). In aerobic cultures, the specific growth rate during the post-diauxic phase (0.10 ± 0.01 h^-1^) was ca. four-fold lower than during the fast growth phase on glucose. As a consequence, the specific growth rate in the hours preceding the onset of SP was ca. three-fold lower in aerobic cultures than in anaerobic cultures. To investigate whether the post-diauxic phase may have ‘conditioned’ aerobic cultures for entry into SP, analysis of aerobic and anaerobic batch cultures was extended to include the growth phases that precede SP.

**Figure 5:**
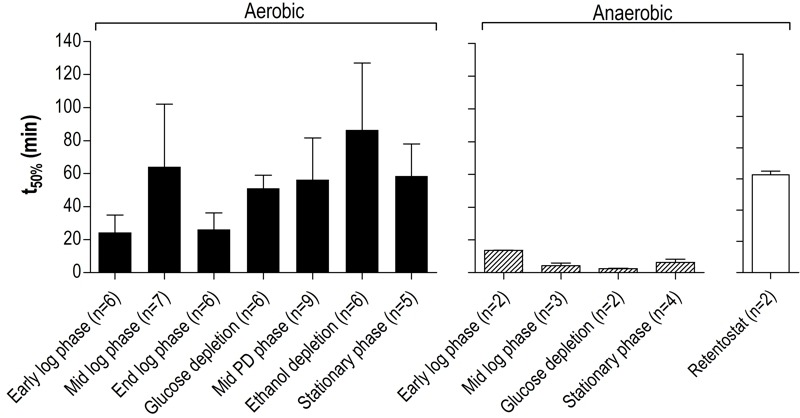
FIGURE 5: Thermotolerance of aerobic and anaerobic cultures during different growth phases. Thermotolerance of cells during different growth phases of aerobic (black bars) and anaerobic (hatched bars) batch cultures of *S. cerevisiae* (Figure 4). The white bar depicts the thermotolerance of *S. cerevisiae* grown for 8 days in anaerobic retentostats 20. Thermotolerance was assayed by monitoring viability during incubation at 53°C and is shown as the incubation time resulting in a 50% decrease in viability (t_50_) (see Materials and Methods for more details). The number of independent culture replicates for each of the growth phases is denoted on the x-axis labels.

A much higher thermotolerance in aerobic cultures was already evident in the exponential growth phase and further increased during the post-diauxic phase, to reach a maximum upon entry into SP (Figure 5). Conversely, thermotolerance of anaerobic cultures did not increase during entry into SP and, consequently, remained much lower than that of aerobic cultures (Figure 5).

**Figure 6:**
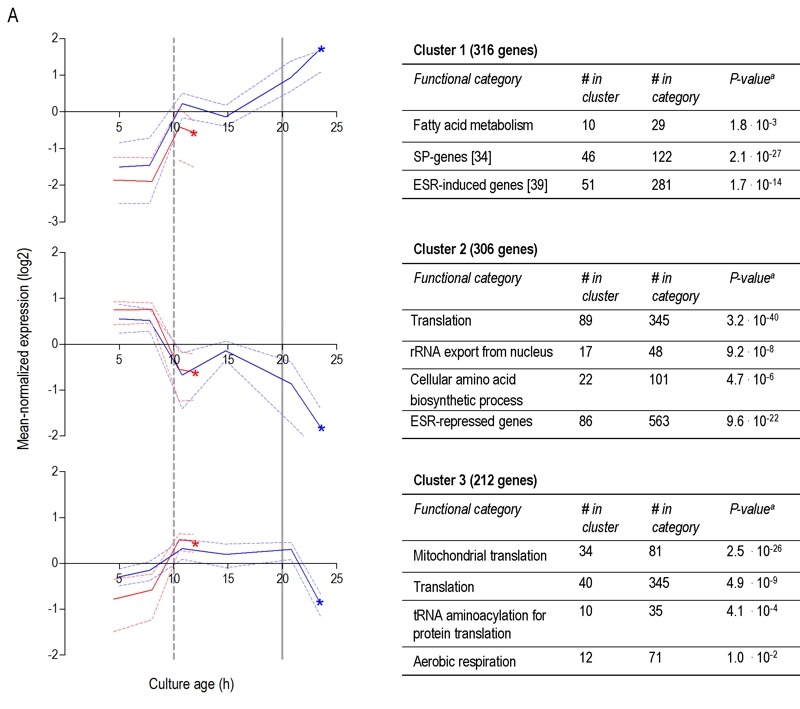

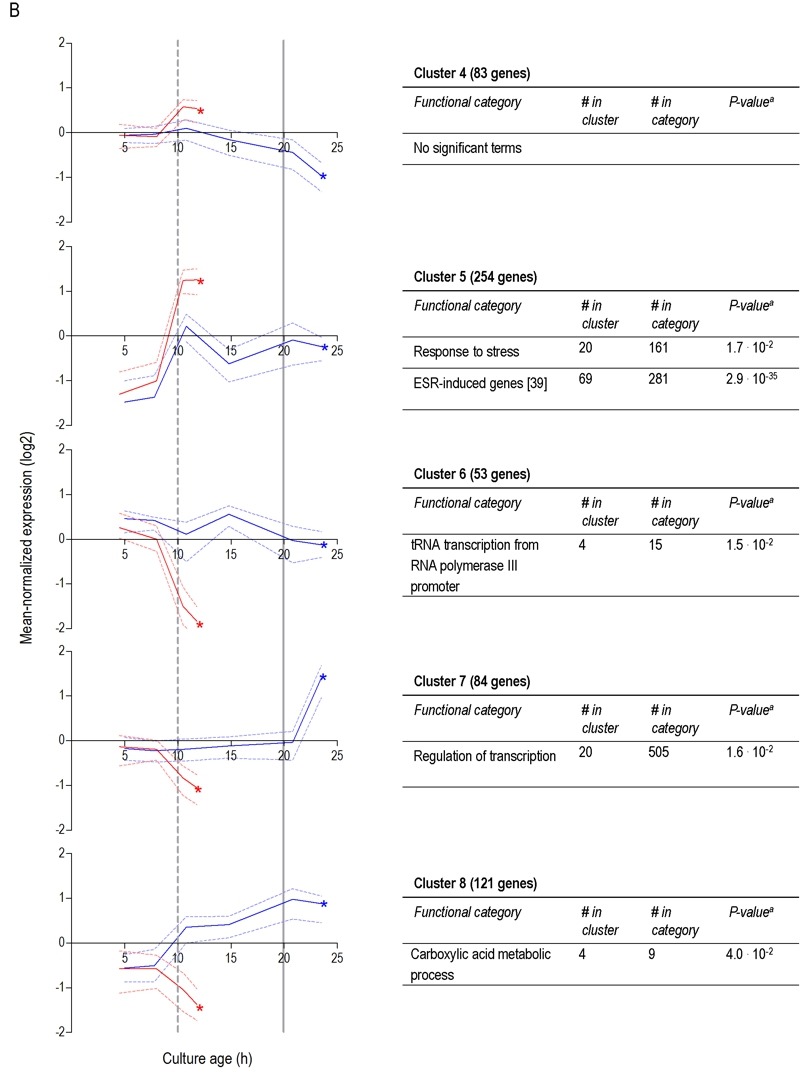
FIGURE 6: Clustering of genes differentially expressed between aerobic and anaerobic SP cultures according to their expression profiles during the growth phases preceding SP. Genes whose differential expression between aerobic and anaerobic SP cultures originated from changes after glucose depletion **(A)**. Clusters of genes whose differential expression between aerobic and anaerobic stationary phase cultures from changes upon glucose exhaustion **(B)**. Each graph presents the expression profiles of genes from aerobic culture** (blue lines)** and anaerobic cultures **(red lines) ** in a particular gene cluster. The solid lines represent the average of the mean-normalized expression of all genes in the cluster. The dashed lines represent the first and third quartile of these mean-normalized expression values, giving information on the scatter in the expression of genes in the cluster. Asterisks (*) indicate the SP samples from anaerobic and aerobic batches. Vertical lines indicate glucose exhaustion **(dashed line) ** and carbon exhaustion (**solid line**, for aerobic cultures only). For each cluster a table reports the overrepresentation of functional categories, including the number of genes in the cluster belonging to a specific functional category (# in cluster), the total number of genes in this functional category (# in category), and the ^a^Bonferroni-corrected P-values that indicate the likelihood of obtaining such enrichment in a random set of genes. Only categories with ^a^Bonferroni-corrected P-value below 0.05 were deemed significant and presented in the tables. More details can be found in Supplemental Table S3.

To further compare the different ‘entry trajectories’ into SP of aerobic and anaerobic batch cultures, transcriptome analyses were performed at different time points during exponential phase, post-diauxic phase (aerobic cultures only) and SP. Genes were grouped in 9 clusters, based on their time-dependent expression profiles in aerobic and anaerobic cultures (Figure 6). A full dataset is available in Supplemental Data (Tables S3 and S4).

**Figure 7:**
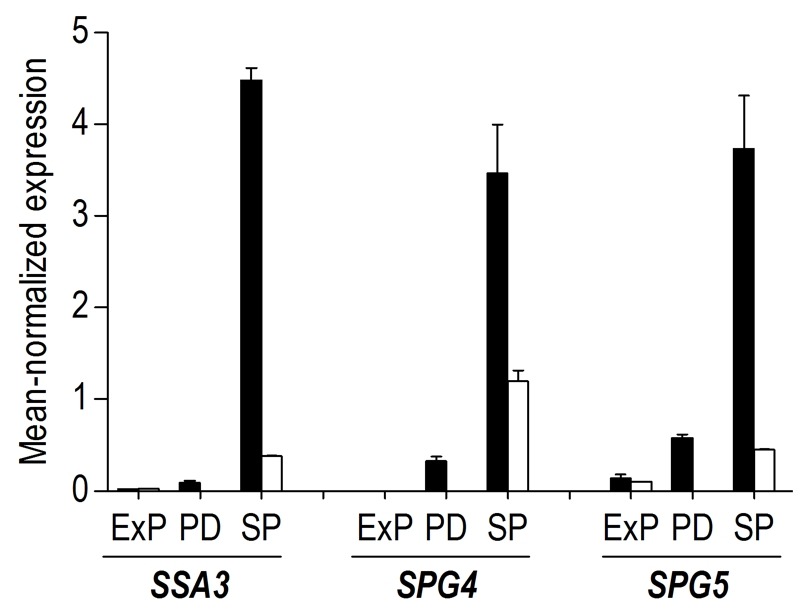
FIGURE 7: Expression levels of the SP-associated genes SSA3, SPG4 and SPG5 during different growth phases in aerobic and anaerobic batch cultures. The mean-normalized expression values during exponential growth on glucose phase (ExP), post-diauxic growth phase (PD, only aerobic cultures) and stationary phase (SP, i.e. 4 hours after exhaustion of exogenous consumable carbon-sources) in aerobic (black bars) and anaerobic (white bars) cultures of *S. cerevisiae* of the genes SSA3, SPG4 and SPG5. Average values of duplicate cultures are shown (± SEM).

Less than one tenth (126 of 1452) of the genes that were differentially expressed in aerobic and anaerobic SP cultures (Table 1) already showed corresponding differences during the mid-exponential growth phase in aerobic and anaerobic cultures. For over half (834 of 1452) of the differentially expressed genes in SP cultures, the differences rose after glucose exhaustion, i.e. during the post-diauxic phase in aerobic cultures (Figure 6A, clusters 1-3). Cluster 1 comprises genes whose expression increased during the aerobic and anaerobic exponential growth phases, with a further increase during the aerobic post-diauxic phase (Figure 6A). Genes involved in fatty acid catabolism were overrepresented in this cluster, as well as genes that were previously shown to be induced in aerobic SP (including *SPG1*, *SPG3*, *SPG4*, *SPG5* and Hsp70*-*family*-*member *SSA3* 34) (Figures 6A and 7). Genes in cluster 2 showed similar transcript levels during the aerobic and anaerobic exponential growth phases on glucose. However, due to a pronounced decrease in expression during the post-diauxic phase in aerobic cultures, expression levels were higher in anaerobic SP cultures than in aerobic SP cultures. This cluster was markedly enriched for genes involved in amino acid synthesis and translation (Figure 6A), suggesting that a down-regulation of protein synthesis occurred during the post-diauxic phase. Cluster 3 comprised of genes whose transcript levels increased during the exponential phase of both aerobic and anaerobic cultures but, subsequently, only decreased in aerobic SP cultures. Cluster 3 showed an overrepresentation of genes involved in mitochondrial translation and respiration (Figure 6A).

For 595 of the ‘oxygen-responsive in SP’ genes listed in Table 1, differences in expression occurred already upon glucose exhaustion (Figure 6B, cluster 4-8). Genes in Cluster 5, characterized by a specific up-regulation upon entry into anaerobic SP, showed an overrepresentation of stress-responsive genes (Figure 6B, cluster 5). Several of these are known to be specifically expressed under anaerobic conditions (e.g., the cell-wall mannoprotein-encoding gene *DAN4* and members of the seripauperin family 40), but

cluster 5 also included heat-shock genes whose expression is not specifically linked to anoxic conditions (e.g. *HSP30* and *SSA4*). Genes that showed a specific downregulation during anaerobic SP, but a constant (Figure 6B, cluster 6) or increased expression in aerobic SP (Figure 6B, clusters 7 and 8) showed an overrepresentation of genes involved in transcription-related processes and carboxylic acid metabolism. The latter of which plays a role in the respiration of exogenous carboxylic acids during the post-diauxic phase (Figure 6B, cluster 8). All 23 genes whose transcript levels were higher under anaerobic conditions, irrespective of the growth phase (Supplemental Table S4), were previously described to be upregulated under anaerobic conditions. The majority (14) of these genes belonged to the seripauperin family 40.

Two clusters (Figure 6, cluster 1 and 5) comprised genes whose transcript levels increased in aerobic as well as in anaerobic batch cultivation, but to different final levels. These clusters were enriched for genes induced by the environmental stress response 39. The extreme differences in thermotolerance of aerobic and anaerobic SP cultures (Figure 5) were therefore only partially mirrored at the transcript level, indicating that factors other than transcriptional reprogramming contribute to these differences.

## DISCUSSION

This study demonstrates a strong impact of oxygen availability on chronological lifespan and stress tolerance in SP batch cultures of *S. cerevisiae* and, thereby, confirms and extends earlier observations on its physiology in aerobic and anaerobic cultures 13,19,47. The CLS of anaerobically grown SP cultures was much shorter than that of their aerobic counterparts and an even more dramatic difference was observed for thermotolerance. As will be discussed below, these differences involved a different conditioning of aerobic and aerobic cultures during the growth phases preceding SP, as well as energetic constraints imposed on yeast cells in anaerobic SP cultures.

### The post-diauxic growth phase enables transcriptional conditioning of aerobic yeast cultures for stationary phase

Our transcriptome data revealed that 57% of the transcriptional differences between aerobic and anaerobic SP cultures originated from transcriptional reprogramming during the aerobic post-diauxic growth phase. Although several well-known hallmark transcripts of SP cultures, previously identified in (semi-) aerobic shake-flask cultures, such as *SPG4*, *SPG5* and *SSA3* 34, showed increased levels in anaerobic SP cultures, their levels did not reach those observed in aerobic cultures (Figure 7). Moreover, many genes involved in biosynthesis were strongly down-regulated during the aerobic post-diauxic phase and SP, but retained expression levels close to those in the exponential growth phase in anaerobic cultures (Figure 6). These transcriptome data are consistent with the hypothesis that the post-diauxic phase in aerobic cultures conditions cells for entry into SP and that, conversely, absence of a post-diauxic phase prevents anaerobic batch cultures from adequately adapting to SP and starvation.

Hormesis could potentially explain the difference in robustness between aerobic and anaerobic cultures. Indeed respiration can generate low levels of ROS and thereby induce stress tolerance via increased expression of stress tolerance genes 13,21. However, among a set of 22 genes encoding enzymes involved in ROS-protective mechanisms 48, including the superoxide dismutase genes *SOD1* and *SOD2*, whose expression is strongly upregulated during exposure to ROS 13,39, only the peroxisomal catalase *CTA1* was higher expressed in aerobic SP cultures. These findings argue against a dominant role of ROS-based hormesis in the acquisition of increased robustness by aerobic SP cultures.

### Caloric restriction: a key factor in conditioning yeast cells for stationary phase and starvation

Thermotolerance is negatively correlated with specific growth rate in *S. cerevisiae* as Lu *et al.* (2009) demonstrated in nutrient-limited chemostat cultures 49. Although these authors did not evaluate the impact of specific growth rate on CLS nor investigate anaerobic growth, they showed that the negative correlation between thermotolerance and growth rate also held in a respiratory-deficient *S. cerevisiae* strain 49. Our data are fully consistent with the hypothesis that the strong reduction of specific growth rate (from 0.39 to 0.10 h^-1^) during transition from fast exponential growth on glucose to the post-diauxic phase in aerobic batch cultures could similarly trigger increased thermotolerance and extended CLS during the starvation phase. We have also recently shown that a gradual decrease of the specific growth rate to near-zero values in glucose-limited retentostats 50 yielded yeast cells with a thermotolerance that is as high as that of aerobic SP cultures (Figure 5), and with an even longer CLS during subsequent starvation 20. The transcriptional reprogramming observed in these anaerobic severely calorie-restricted cultures 51 strongly resembled the transcriptome changes observed in the present study for aerobic cultures entering SP and proteome analysis showed increased levels of proteins involved in stress resistance 52. Deletion of Rim15, a kinase under control of several nutrient signaling pathways 53, strongly reduced the acquisition of robustness in both anaerobic and aerobic calorie-restricted cultures 54,55, suggesting a strong role for nutrient signaling independent of oxygen availability.

The present study, combined with our previous retentostat studies, therefore clearly demonstrates that prior conditioning by a period of caloric restriction (e.g. by slow growth during the aerobic post-diauxic phase or in extremely glucose-limited cultures) is a prerequisite for acquisition of a prolonged CLS by non-growing, starving cultures of *S. cerevisiae*. This conclusion, which supports earlier proposals based on starvation experiments by Thomsson *et al*. 47,56, has important implications for the design and interpretation of yeast studies on chronological aging, for example when such studies involve mutants that are impeded in energy metabolism.

### The low thermotolerance of exponentially growing anaerobic cultures does not correlate with expression of heat shock genes 

Although both aerobic and anaerobic, non-growing yeast cultures can acquire a similar thermotolerance by an appropriate preceding conditioning phase, a drastic difference was observed in the thermotolerance of exponentially growing aerobic and anaerobic cultures (Figure 5). The similar adenylate energy charge and intracellular ATP concentrations of aerobic and anaerobic cultures during exponential growth on glucose appear to rule out cellular energy status as a major cause of this difference. Since the high temperature (53°C) during the thermotolerance assays precludes *de novo* synthesis of mRNA or protein synthesis 57, this difference must already be expressed in the batch cultures themselves. Transcript levels of genes that were previously implicated in heat-shock resistance (including *HSP* genes 58 such as *HSP104* 59, *HSP26* 60, *HSP12* 61, *SSA3* 62) were similar during the exponential growth phase on glucose in aerobic and anaerobic cultures. Moreover, of 59 genes identified as essential for heat-shock survival by Gibney and coworkers 57, only one gene was differentially expressed, *LIA1*, and showed a higher transcript level in anaerobic cultures.

Oxygen availability strongly influences sterol and unsaturated fatty acid composition of yeast cells 19, especially because these compounds have to be added to growth media as anaerobic growth factors 43. These differences in membrane composition might partially explain the observed differences in thermotolerance between aerobically and anaerobically grown *S. cerevisiae *cells*. *The hypothesis that membrane composition is a key determinant in thermotolerance of *S. cerevisiae* 63,64 is consistent with a recent study, in which the acquisition of increased thermotolerance by laboratory-evolved strains was shown to be caused by changes in their sterol composition 65.

### A low energy status of anaerobic SP cultures limits metabolic flexibility

Consistent with earlier reports 19,66, anaerobic batch cultures of *S. cerevisiae* displayed a substantially higher glycogen content than aerobic cultures. However, after the onset of SP, anaerobic cultures showed a much faster decrease of the adenylate energy charge. This difference can be attributed to several factors. Firstly, since *S. cerevisiae* cannot derive metabolic energy from lipids and amino acids in the absence of oxygen 67, anaerobic cultures are entirely dependent on glycogen and trehalose as energy storage compounds and anaerobic catabolism of these storage carbohydrates yields less ATP than respiration. The estimated ATP synthesis rate from anaerobic glycogen dissimilation of ca. 5 µmol per g biomass dry weight per hour during the first day in SP (based on a maximum ATP yield of 3 ATP per glucose residue 20), was two orders of magnitude lower than the cellular ATP demand for maintenance estimated from chemostat and retentostat cultures (m_ATP_ = 1 mmol ATP per g biomass dry weight per hour 50). A similar extreme reduction of ATP turnover rates was observed when anaerobic retentostat cultures were switched to carbon starvation 20. Together, these observations indicate that an extremely low ATP turnover is an intrinsic feature of anaerobic, starving yeast cultures. In addition to this extreme low ATP-turnover, it is even conceivable that the apparent inability of anaerobic batch cultures to efficiently down-regulate energy-consuming processes, including protein synthesis, the single most expensive biosynthetic process in living cells 68,69, may have exacerbated the fast decline of their energy status after entry into SP (Figure 3).

The low energy status of cells may at the same time have put strong constraints on these energy consuming processes. Proteome analyses should reveal whether the increased transcription of *HSP* genes, implicated in thermotolerance, which took place late in the exponential growth phase (Figure 6), was too late to enable synthesis of the corresponding proteins before the decline in cellular energy status in anaerobic SP cultures. Such a scenario would explain the discrepancy between the oxygen-independent upregulation of these genes (with notable exception of *SSA3*) and the absence of increased thermotolerance in anaerobic SP cultures.

Taken together, the results from the present study indicate that, in the short time lapse between the moment at which anaerobic cultures sense that glucose reaches critically low levels and the actual exhaustion of glucose, they lack the time and resources to perform the energy-intensive remodeling of their transcriptome and proteome required to robustly face starvation. Our data are therefore entirely consistent with the notion that the low CLS and thermotolerance of anaerobic SP cultures, in comparison with aerobic cultures, is due to the absence of a proper conditioning phase and a limited metabolic flexibility due to a lower cellular energy status.

### Outlook

Many studies in which SP yeast cultures are used as a model system to investigate aspects of aging, still rely on shake-flask cultures. Due to their low and poorly controlled oxygen-transfer capacity, the aeration status of shake-flask cultures is generally unclear. The strong impact of oxygen availability on aging-related characteristics 14 underlines the value of controlled cultivation techniques, e.g. in bioreactors, including batch, chemostat and retentostat cultures 50,70,71,72 or flow-through cells 73, in yeast-based aging studies. In particular, the use of anaerobic cultures as a model offers interesting possibilities to clarify the role of respiration and ROS in aging, apoptosis and longevity.

The short life span and low robustness of anaerobic SP cultures of *S. cerevisiae* is directly relevant for industrial applications. Robustness of SP cultures is especially important for processes in which biomass from anaerobic batch cultures is recycled, e.g. in industrial bioethanol production and beer brewing 74,75. Clearly, results from (semi-)aerobic shake-flask cultures cannot be used to predict the performance of such anaerobic processes and improvement of robustness in these industrial processes will have to be based on studies in anaerobic systems.

*Saccharomyces* yeasts have the capability, rare among eukaryotes, to grow fast in the complete absence of oxygen and it is often assumed that they are well adapted to anaerobic environments 30,76,77. While the natural habitat of *S. cerevisiae* is still a matter of debate 77, lower biomass concentrations frequently encountered in natural environments combined with the low affinity of yeast glucose transporters 78 may lead to a transition into SP that is sufficiently slow to enable acquisition of longevity and robustness under anaerobic conditions. Further research is therefore needed to investigate the ecological relevance of this laboratory study.

## MATERIALS AND METHODS

### Strains and cultivation

The prototrophic *Saccharomyces cerevisiae* strain CEN.PK113-7D (*MAT*a *MAL*2-8c *SUC*2) used in this study is a congenic member of the CEN.PK family 79,80. Stock cultures were grown at 30°C in shake flasks containing yeast extract (1% w/v), peptone (2% w/v) and dextrose (2% w/v) (YPD) medium. Glycerol, final concentration 20% (v/v), was added to overnight cultures and 1 mL aliquots were stored at -80°C.

Previously described synthetic medium 81 was used with 20 g/L glucose as sole carbon-source and 0.2 g/L antifoam Emulsion C (Sigma, St. Louise, USA). In case of anaerobic cultivations, the medium was supplemented with anaerobic growth factors ergosterol (10 mg/L) and Tween 80 (420 mg/L) dissolved in ethanol. Inocula for batch fermentations consisted of 100 mL yeast culture grown overnight to an OD_660_ of 4 in synthetic medium with 20 g/L glucose. Aerobic and anaerobic batch fermentations were carried out at 30°C in 2 L bioreactors (Applikon, Schiedam, The Netherlands), with a working volume of 1.4 L. Cultures were stirred at 800 rpm and sparged at a flow-rate of 700 mL/min with either dried air or nitrogen gas (< 10 ppm oxygen, Linde Gas Benelux, The Netherlands). The bioreactors were equipped with Norprene tubing (Saint-Gobain Performance Plastics, Courbevoie, France) and Viton O-rings (Eriks, Alkmaar, The Netherlands) to minimize diffusion of oxygen. During aerobic cultivations, dissolved oxygen levels remained above 40% of the initial saturation level as measured by Clark electrodes (Mettler Toledo, Greifensee, Switzerland). The culture pH was maintained at 5.0 by automatic addition of 2 M KOH and 2 M H_2_SO_4_.

### Analysis of biomass, metabolites, substrate and exhaust gas

Biomass concentration as culture dry weight was determined as described previously 82.

For substrate and extracellular metabolite concentration determination, culture supernatants were obtained by centrifugation of culture samples (3 min at 20.000 g) and analysed by high-performance liquid chromatograph (HPLC) analysis on a Waters Alliance 2690 HPLC (Waters, Milford, MA) equipped with a Bio-Rad HPX 87H ion exchange column (BioRad, Veenendaal, The Netherlands), operated at 60°C with 5 mM H_2_SO_4_ as the mobile phase at a flow rate of 0.6 ml/min. Detection was by means of a dual-wavelength absorbance detector (Waters 2487) and a refractive index detector (Waters 2410).

The exhaust gas from batch cultures was cooled with a condenser (2°C) and dried with a PermaPure Dryer (model MD 110-8P-4; Inacom Instruments, Veenendaal, the Netherlands) prior to online analysis of carbon dioxide and oxygen with a Rosemount NGA 2000 Analyser (Baar, Switzerland).

### Colony forming units

To determine culture viability, small aliquots of culture broth were taken from the reactor and cells were counted on a Z2 Coulter Counter (Beckman Coulter, Woerden, Netherlands) equipped with a 50 µm orifice (Multisizer II, Beckman Coulter, Woerden, Netherlands). Cells were diluted in 0.1% peptone and 100 µL suspensions containing approximately 30, 300 and 3000 cells were plated on yeast extract (1% w/v), peptone (2% w/v) and dextrose (0.5% w/v) (YPD) agar plates and incubated at 30°C for at least 3 days before counting the colonies. CFU was calculated as the number of colonies formed divided by the number of plated cells.

### Thermotolerance assay

Cells from culture broth were counted with a Z2 Coulter Counter and diluted in pre-warmed (53°C) isotone diluent II (Beckman Coulter, Woerden, Netherlands) to yield 50 mL cell suspensions with a density of 1∙10^7^ cells/mL. Cell suspensions were incubated in a waterbath at 53°C and 4 mL aliquots were sampled in 10 min intervals. Samples were cooled on ice and assayed for viability using the *Funga*Light 5-CFDA, AM (acetoxymethyl ester 5-carboxyfluorescein diacetate)/propidum iodide (PI) yeast viability kit (Invitrogen, Carlsbrad, CA) by counting 10,000 cells on a Cell Lab Quanta SC MPL flow cytometer (Beckman Coulter, Woerden, Netherlands) as described previously 20. 5-CFDA, AM is a cell-permeant substrate for intracellular non-specific esterase activity. Hydrolytic cleavage of the lipophilic blocking acetoxymethyl and diacetate groups of 5-CFDA, AM results in a green fluorescent signal in metabolically active cells. Propidium iodide intercalates with DNA in cells with a compromised cell membrane, which results in red fluorescence. Cells stained with PI were considered dead cells. For each independent sample, the t_50_ value (the time after which 50% of the initial viable population was dead) was estimated by fitting the viability data with a sigmoidal dose-response curve in Graphpad Prism 4.03. Both measurements of viability, i.e. metabolic activity based on 5-CFDA, AM and membrane integrity based on PI gave similar t_50_-values, therefore only results based on PI are shown.

### Storage carbohydrate measurements

1.2 mL culture broth was quenched in 5 mL of cold methanol (-40°C) using a rapid sampling setup described previously 83, mixed and subsequently pelleted (4,400 *g*, 5 min) at -19°C. Cells were washed with 5 mL of cold methanol (-40^o^C) and pellets stored at -80°C. Pellets were resuspended in Na_2_CO_3_ (0.25 M) and further processed according to a previously described procedure 84.

### Fatty acids measurements

Culture volumes corresponding to 50 mg biomass were sampled on ice, centrifuged (10,000 *g*, 10 min at 4°C), washed twice, resuspended in 5 ml ice-cold water and stored at -20°C. Lipid extraction was performed as described previously 85. Aliquots of 0.15 mL were added to 15 mL tubes and 1.5 mL of a mixture of concentrated HCl and 1-propanol (1:4) and 1.5 mL of dichlorethane were added. 400 μg of myristic acid (a 15:0 fatty acid) was included as internal standard. Samples were incubated at 100°C for 2 h. Subsequently, 3 mL of water was added to cooled samples. 1 mL of the organic phase was filtered over water-free sodium sulfate into GC vials. The fatty acid propyl esters in the organic phase were analyzed by gas chromatography (model 6890N, Agilent, U.S.A.) using a DB-wax column (length, 30 m; inside diameter, 0.25 mm; film thickness, 0.25 μm; J&W Scientific, Folsom, CA) and helium as the carrier gas. The sample volume was 1 μL, and the split was set to 1:20. The injection temperature was 230°C, and the following temperature gradient was used: 120°C at the start, increasing at a rate of 10°C/min up to 240°C, and then 240°C for 8 min. The fatty acid propyl esters were detected using a flame ionization detector at 250°C.

### Analysis of intracellular adenosine-phosphate concentrations 

Samples for internal metabolite analysis were obtained by rapid sampling 83. 1.2 mL of culture broth was rapidly quenched into 5 mL of 100% methanol, pre-cooled to -40°C. Samples were washed with cold methanol and extracted with boiling ethanol. Intracellular AMP and ADP were determined enzymatically, using a previously described assay based on myokinase, pyruvate kinase and lactate dehydrogenase reactions 86. Assays were performed in white, flat bottom 96-well microtiter plates (Corning Inc., USA). NADH fluorescence was measured in a TECAN GENios Pro microtiterplate reader (Tecan, Männedorf, Switzerland) as previously described 83. Intracellular ATP was also assessed enzymatically. The assay contained 115 mM triethanolamine (pH 7.6), 11.5 mM MgSO_4_x7H_2_O, 1.15 mM NADP^+^ per well including sample extract, total volume was 205 µL per well. The reaction to measure ATP was initiated by adding 12 mM glucose and 30 U hexokinase (Sigma-Aldrich Chemie B.V, Zwijndrecht, The Netherlands). Assays were performed in black, flat bottom 96-well microtiter plates (Corning Inc., USA). NADPH fluorescence was measured in a TECAN GENios Pro microtiterplate reader. The adenylate energy charge was calculated according to the previously described 42 equation:

**Figure:**
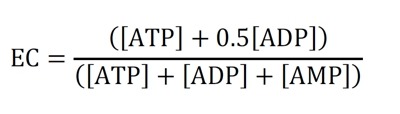


### Transcriptome analysis

Independent duplicate aerobic and anaerobic batch cultures were sampled at six and four different time points respectively (see Figure 4A and 4B) for microarray analysis, resulting in a total dataset of 20 microarrays. Sampling from batch cultures for transcriptome analysis was performed using liquid nitrogen for rapid quenching of mRNA turnover 87. Prior to RNA extraction, samples were stored in a mixture of phenol/chloroform and TEA buffer at -80°C. Total RNA extraction, isolation of mRNA, cDNA synthesis, cRNA synthesis, labelling and array hybridization was performed as previously described 88. The quality of total RNA, cDNA, aRNA and fragmented aRNA was checked using an Agilent Bioanalyzer 2100 (Agilent Technologies, Santa Clara, CA). Hybridization of labelled fragmented aRNA to the microarrays and staining, washing and scanning of the microarrays was performed according to Affymetrix instructions (EukGE_WS2v5).

The 6383 yeast open reading frames were extracted from the 9335 transcript features on the YG-S98 microarrays. To allow comparison, all expression data were normalized to a target value of 240 using the average signal from all gene features 89. The microarray data used in this study are available via GEO series accession number GSE69485. To eliminate variation in genes that are not expressed, genes with expression values below 12 were set to 12 and the gene features for which the maximum expression was below 20 for all 20 arrays were discarded 51. The average deviation of the mean transcript data of replicate batches was ca. 11%, similar to the reproducibility usually observed in replicate steady state chemostat cultures 90. The expression of housekeeping genes *ACT1, PDA1*, *TFC1, ALG9, TAF10 *and* UBC6* 91 remained stable for both conditions and all sample points (average coefficient of variation 17% ± 5%).

To identify genes that were differentially expressed between aerobic and anaerobic SP cultures, a pairwise comparison was performed between aerobic samples taken at time point 6 (Figure 4A) and anaerobic samples taken at time point 4 (Figure 4B) as previously described 89. Similarly, genes differently expressed during growth on glucose under aerobic or anaerobic conditions were identified through a pairwise comparison of aerobic and anaerobic samples taken at time point 1 (Figure 4A and 4B). Differences with adjusted P-values lower than 0.05 and a fold difference of 2 or higher were considered statistically significant. Time-dependent expression profiles of selected genes were clustered according to optimal k-means clustering using positive correlation as distance metric (Expressionist Pro version 3.1, Genedata, Basel, Switzerland) resulting in an optimal number of 9 clusters. For display of time-dependent transcript levels, expression values were normalized per gene by dividing single expression values by the average expression value of both conditions and all time points. Mean values of these average-normalized values for all genes in each cluster are shown, as well as the first and third quartile of average-normalized values.

Gene expression clusters were analysed for overrepresentation of functional annotation categories of the Gene Ontology (GO) database (http://www.geneontology.org , based on a hypergeometric distribution analysis tool 92. Additional categories describing genes expressed in SP cultures 34, genes commonly induced by several environmental stresses 39 or essential for heat-shock survival 57 were extracted from the respective references.

## SUPPLEMENTAL MATERIAL

Click here for supplemental data file.

All supplemental data for this article are also available online at http://microbialcell.com/researcharticles/oxygen-availability-strongly-affects-chronological-lifespan-and-thermotolerance-in-batch-cultures-of-saccharomyces-cerevisiae/.
