# Supplementary material for: Oxygen availability strongly affects chronological lifespan and thermotolerance in batch cultures of Saccharomyces cerevisiae
Source: Microb Cell. 2015 Oct 21;2(11):429–44. doi: 10.15698/mic2015.11.238 (PMC5349206; doi:10.15698/mic2015.11.238)
Supplement: Supplementary file 1 [file mic-02-429-s01.pdf]

Table S1 Genes with significantly different expression in aerobic or anaerobic stationary phase cultures.
Fold change cut-off of 2.0 and an adjusted p-value cut-off of 0.05 were used to determine significance.
Cluster numbers refer to the clusters displayed in figure 6 and 7 of the manuscript.

Genes higher expressed in anaerobic SP

Probeset id	Systematic name	Standard name	Fold change	Adjusted P	Cluster
10852_at	YJR150C	DAN1	48,3	3,25E-04	9
4876_at	YGR131W	---	17,6	0,00E+00	5
8526_at	YOR009W	TIR4	17,3	0,00E+00	9
8528_at	YOR011W	AUS1	16,4	0,00E+00	3
10861_s_at	YJR159W	SOR1 /// SOR2	15,5	0,00E+00	3
10976_at	YJR047C	ANB1	14,9	0,00E+00	2
6012_at	YDR453C	TSA2	14,1	0,00E+00	5
5537_at	YER188W	---	14,0	0,00E+00	3
4811_at	YGR201C	---	13,0	0,00E+00	5
9290_f_at	YMR325W	PAU19 /// PAU21 /// P	11,9	0,00E+00	9
9947_i_at	YLR367W	RPS22B	11,7	1,32E-10	2
5711_at	YER011W	TIR1	11,6	6,79E-09	9
8862_at	YNR014W	---	11,4	0,00E+00	5
6798_f_at	YCR104W	PAU19 /// PAU21 /// P	10,7	0,00E+00	9
9716_at	YML083C	---	10,3	0,00E+00	9
9293_at	YML058w-a	HUG1	10,0	0,00E+00	9
4114_at	YIL011W	TIR3	9,9	1,74E-12	2
10289_f_at	YLR037C	PAU23	9,8	0,00E+00	5
4076_f_at	YIR041W	PAU15 /// PAU16	9,1	0,00E+00	9
10789_f_at	YKL224C	PAU15 /// PAU16	8,2	0,00E+00	5
4100_at	YIR019C	MUC1	8,0	0,00E+00	2
6486_at	YDL021W	GPM2	7,9	0,00E+00	5
5942_at	YDR518W	EUG1	7,7	0,00E+00	2
7645_at	YPR113W	PIS1	7,7	0,00E+00	2
4322_at	YHR210C	---	7,3	0,00E+00	4
8546_at	YOR028C	CIN5	7,1	0,00E+00	5
5940_at	YDR516C	EMI2	6,7	0,00E+00	3
10375_at	YLL055W	YCT1	6,6	0,00E+00	3
10909_at	YJR116W	---	6,6	0,00E+00	2
5786_f_at	YEL049W	PAU2	6,5	0,00E+00	5
9388_at	YMR250W	GAD1	6,4	0,00E+00	5
8023_at	YPL272C	---	6,2	5,77E-11	9
4472_s_at	YHR043C	DOG1 /// DOG2	6,2	0,00E+00	4
8378_at	YOR176W	HEM15	6,2	0,00E+00	3
10412_f_at	YLL064C	PAU1 /// PAU14 /// PA	6,1	0,00E+00	9
8784_s_at	YNR073C	DSF1	6,1	0,00E+00	5
10361_f_at	YLL025W	PAU17	6,0	0,00E+00	5
7604_at	YPR160W	GPH1	5,9	0,00E+00	5
11177_at	YJL161W	FMP33	5,9	0,00E+00	5
4442_at	YHR104W	GRE3	5,9	0,00E+00	5
4043_s_at	YIL162W	SUC2	5,8	0,00E+00	5
5971_at	YDR502C	SAM2	5,7	0,00E+00	2
6413_at	YDR044W	HEM13	5,7	0,00E+00	2
10681_at	YKL103C	LAP4	5,5	0,00E+00	3
8786_f_at	YNR076W	PAU1 /// PAU14 /// PA	5,5	0,00E+00	9
5117_at	YGL125W	MET13	5,5	0,00E+00	4
5251_f_at	YGL261C	PAU10 /// PAU11 /// P	5,5	0,00E+00	9
7964_at	YPL196W	OXR1	5,4	0,00E+00	5
5751_at	YEL039C	CYC7	5,4	0,00E+00	5
10722_at	YKL151C	---	5,3	0,00E+00	5
8720_f_at	YOL161C	PAU18 /// PAU20 /// P	5,3	0,00E+00	9
5016_at	YGL001C	ERG26	5,3	0,00E+00	2
3937_g_at	SUC4	SUC2	5,3	0,00E+00	5
5575_at	YER134C	---	5,2	0,00E+00	4
4200_at	YIL111W	COX5B	5,2	0,00E+00	5
4573_at	YHL035C	VMR1	5,2	0,00E+00	5
9365_at	YMR271C	URA10	5,2	0,00E+00	5
5013_at	YGL004C	RPN14	5,2	0,00E+00	5
6837_at	YCR048W	ARE1	5,2	0,00E+00	2
4392_at	YHR143W	DSE2	5,1	0,00E+00	4
8003_at	YPL247C	---	5,1	0,00E+00	5

4947_at	YGR066C	---	5,0	0,00E+00	5
8140_at	YOR386W	PHR1	5,0	0,00E+00	5
5752_at	YEL038W	UTR4	5,0	0,00E+00	5
10158_at	YLR178C	TFS1	5,0	0,00E+00	5
9059_at	YNL200C	---	5,0	0,00E+00	5
9865_f_at	YLR461W	PAU10 /// PAU11 /// P.	5,0	0,00E+00	9
6057_at	YDR406W	PDR15	5,0	0,00E+00	5
4943_at	YGR062C	COX18	5,0	0,00E+00	3
9481_at	YMR169C	ALD3	5,0	0,00E+00	5
4717_at	YGR287C	---	4,9	0,00E+00	5
6725_at	YDL227C	HO	4,9	0,00E+00	2
4563_f_at	YHL046C	PAU1 /// PAU10 /// PA	4,9	0,00E+00	9
8303_at	YOR237W	HES1	4,8	0,00E+00	9
11251_s_at	YJL221C	FSP2	4,7	0,00E+00	5
9691_i_at	YML063W	RPS1B	4,7	1,05E-06	2
6178_at	YDR304C	CPR5	4,6	0,00E+00	4
7074_f_at	YBR301W	PAU12 /// PAU24	4,6	0,00E+00	5
8264_at	YOR288C	MPD1	4,6	0,00E+00	4
7351_at	YBR037C	SCO1	4,6	0,00E+00	3
5410_f_at	YFL020C	PAU5	4,5	0,00E+00	5
9203_at	YNL327W	EGT2	4,5	0,00E+00	4
4804_at	YGR194C	XKS1	4,5	0,00E+00	5
5253_g_at	YGL259W	YPS5 /// YPS6	4,5	0,00E+00	5
5034_at	YGL028C	SCW11	4,5	0,00E+00	4
4732_at	YGR256W	GND2	4,5	0,00E+00	5
5643_at	YER070W	RNR1	4,4	2,07E-09	2
11254_at	YJL218W	---	4,4	0,00E+00	4
7722_at	YPR015C	---	4,4	0,00E+00	5
4112_at	YIL013C	PDR11	4,3	0,00E+00	5
4970_at	YGR044C	RME1	4,3	0,00E+00	3
7712_at	YPR047W	MSF1	4,3	0,00E+00	3
6539_at	YDL059C	RAD59	4,3	0,00E+00	4
9355_at	YMR261C	TPS3	4,3	0,00E+00	4
10060_at	YLR258W	GSY2	4,3	0,00E+00	5
4530_at	YHR011W	DIA4	4,2	0,00E+00	3
6270_at	YDR213W	UPC2	4,2	0,00E+00	2
4992_at	YGR019W	UGA1	4,2	0,00E+00	5
5357_at	YFR015C	GSY1	4,2	0,00E+00	5
9442_s_at	YMR173W	DDR48	4,2	0,00E+00	5
4723_f_at	YGR294W	PAU10 /// PAU11 /// P.	4,1	0,00E+00	9
6704_at	YDL204W	RTN2	4,1	0,00E+00	5
3907_f_at	YAL068C	PAU1 /// PAU10 /// PA	4,1	0,00E+00	9
7337_at	YBR024W	SCO2	4,1	0,00E+00	5
4336_at	YHR176W	FMO1	4,1	0,00E+00	3
11051_at	YJL017W	---	4,1	0,00E+00	5
4088_at	YIR007W	---	4,1	0,00E+00	5
6110_at	YDR368W	YPR1	4,0	0,00E+00	4
11142_at	YJL105W	SET4	4,0	2,18E-09	5
5941_at	YDR517W	GRH1	4,0	0,00E+00	3
10161_g_at	YLR180W	SAM1 /// SAM2	4,0	0,00E+00	2
3403_f_at	YDR542W	PAU10 /// PAU11 /// P.	4,0	0,00E+00	9
7254_at	YBR117C	TKL2	4,0	0,00E+00	5
4280_f_at	YIL176C	PAU1 /// PAU10 /// PA	4,0	0,00E+00	9
9904_at	YLR413W	---	3,9	8,38E-09	2
10860_s_at	YJR158W	HXT13 /// HXT15 /// H)	3,9	0,00E+00	5
11249_f_at	YJL223C	PAU1 /// PAU10 /// PA	3,9	0,00E+00	9
5301_at	YFR047C	BNA6	3,9	0,00E+00	3
9729_at	YML070W	DAK1	3,9	0,00E+00	5
10050_at	YLR290C	---	3,9	0,00E+00	5
6109_at	YDR367W	KEI1	3,9	0,00E+00	2
10911_at	YJR118C	ILM1	3,9	0,00E+00	2
6703_at	YDL205C	HEM3	3,9	0,00E+00	4
5067_at	YGL040C	HEM2	3,9	0,00E+00	3
4832_at	YGR177C	ATF2	3,9	3,93E-07	2
7264_at	YBR086C	IST2	3,9	0,00E+00	4
8467_at	YOR086C	TCB1	3,8	0,00E+00	3
10768_at	YKL194C	MST1	3,8	5,92E-13	3

9418_at	YMR193W	MRPL24	3,8	0,00E+00	3
8647_at	YOL096C	COQ3	3,8	0,00E+00	3
9482_g_at	YMR169C	ALD2 /// ALD3	3,8	0,00E+00	5
7236_at	YBR145W	ADH5	3,8	0,00E+00	4
10755_at	YKL164C	PIR1	3,8	0,00E+00	4
7686_at	YPR066W	UBA3	3,8	0,00E+00	5
10068_i_at	YLR264W	RPS28B	3,7	1,70E-08	2
7632_i_at	YPR102C	RPL11A /// RPL11B	3,7	4,47E-04	2
5093_at	YGL104C	VPS73	3,7	0,00E+00	5
10998_at	YJR021C	REC107	3,7	0,00E+00	5
4512_at	YHR038W	RRF1	3,7	0,00E+00	3
8727_at	YOL153C	---	3,7	0,00E+00	5
4912_at	YGR076C	MRPL25	3,7	0,00E+00	3
10660_at	YKL080W	VMA5	3,7	0,00E+00	2
9445_at	YMR175W	SIP18	3,7	0,00E+00	5
11259_at	YJL213W	---	3,7	0,00E+00	5
6593_at	YDL093W	PMT5	3,6	0,00E+00	2
9925_at	YLR390W	ECM19	3,6	0,00E+00	3
7321_at	YBR053C	---	3,6	0,00E+00	5
4994_at	YGR021W	---	3,6	0,00E+00	3
6380_at	YDR100W	TVP15	3,6	0,00E+00	3
6890_at	YCR012W	PGK1	3,6	1,17E-12	2
7263_at	YBR085W	AAC3	3,6	0,00E+00	2
10101_at	YLR209C	PNP1	3,6	0,00E+00	3
9162_at	YNL277W	MET2	3,5	0,00E+00	4
5615_at	YER087W	AIM10	3,5	0,00E+00	3
4213_at	YIL098C	FMC1	3,5	0,00E+00	3
9953_at	YLR372W	SUR4	3,5	4,02E-12	2
8843_at	YNR040W	---	3,5	0,00E+00	3
6488_at	YDL019C	OSH2	3,5	0,00E+00	3
6139_at	YDR353W	TRR1	3,5	0,00E+00	2
7699_at	YPR036W	VMA13	3,5	0,00E+00	2
11060_at	YJL052W	TDH1	3,5	0,00E+00	5
9727_at	YML072C	TCB3	3,5	0,00E+00	4
6204_at	YDR284C	DPP1	3,5	0,00E+00	3
9097_at	YNL252C	MRPL17	3,5	0,00E+00	3
4541_at	YHL021C	AIM17	3,4	0,00E+00	5
6351_at	YDR116C	MRPL1	3,4	0,00E+00	3
9008_at	YNL160W	YGP1	3,4	0,00E+00	5
6213_at	YDR248C	---	3,4	0,00E+00	3
7253_g_at	YBR116C	TKL2	3,4	0,00E+00	5
7582_at	YPR184W	GDB1	3,4	0,00E+00	5
7363_at	YBR003W	COQ1	3,4	0,00E+00	3
11355_at	YAL012W	CYS3	3,4	0,00E+00	2
6088_at	YDR391C	---	3,4	0,00E+00	5
8505_at	YOR032C	HMS1	3,4	0,00E+00	4
5556_at	YER163C	---	3,4	0,00E+00	4
10300_f_at	YLR048W	RPS0B	3,4	1,74E-07	2
4521_at	YHR004C	NEM1	3,4	0,00E+00	5
6485_at	YDL022W	GPD1	3,4	0,00E+00	3
11287_f_at	YAR020C	PAU7	3,4	0,00E+00	5
6560_at	YDL082W	RPL13A	3,4	1,94E-09	2
4768_at	YGR248W	SOL4	3,4	0,00E+00	5
9532_at	YMR090W	---	3,4	0,00E+00	5
4428_at	YHR092C	HXT4	3,4	6,98E-04	2
8988_at	YNL134C	---	3,4	0,00E+00	5
10303_at	YLR049C	---	3,4	0,00E+00	3
11289_at	YAR027W	UIP3	3,3	5,71E-12	5
9870_s_at	YLR312W-A	MRPL15	3,3	0,00E+00	3
4513_at	YHR039C	MSC7	3,3	0,00E+00	4
9567_at	YMR081C	ISF1	3,3	0,00E+00	5
6456_at	YDR041W	RSM10	3,3	0,00E+00	3
5240_at	YGL229C	SAP4	3,3	0,00E+00	3
5919_at	YDR540C	IRC4	3,3	1,91E-04	5
9422_at	YMR195W	ICY1	3,3	0,00E+00	3
4126_at	YIL045W	PIG2	3,3	0,00E+00	5
7735_at	YPR028W	YOP1	3,3	0,00E+00	4

6552_at	YDL089W	NUR1	3,3	0,00E+00	5
7237_at	YBR146W	MRPS9	3,3	0,00E+00	3
4951_at	YGR070W	ROM1	3,3	0,00E+00	5
7256_at	YBR120C	CBP6	3,3	0,00E+00	3
10721_at	YKL152C	GPM1	3,3	0,00E+00	2
5025_at	YGL037C	PNC1	3,3	0,00E+00	5
8412_at	YOR120W	GCY1	3,3	0,00E+00	5
6568_f_at	YDL075W	RPL31A	3,3	4,44E-07	2
8474_at	YOR092W	ECM3	3,3	0,00E+00	2
10566_at	YKR003W	OSH6	3,3	0,00E+00	3
10563_at	YKL001C	MET14	3,3	0,00E+00	2
8897_at	YNL045W	LAP2	3,3	0,00E+00	5
9777_at	YML110C	COQ5	3,3	0,00E+00	3
6929_at	YCL047C	---	3,2	1,89E-10	2
6483_at	YDL024C	DIA3	3,2	0,00E+00	5
8574_at	YOL033W	MSE1	3,2	0,00E+00	3
8211_at	YOR321W	PMT3	3,2	0,00E+00	2
6427_at	YDR058C	TGL2	3,2	5,92E-13	4
9468_at	YMR157C	AIM36	3,2	0,00E+00	3
10652_at	YKL087C	CYT2	3,2	0,00E+00	3
8879_at	YNL016W	PUB1	3,2	0,00E+00	2
7144_at	YBR234C	ARC40	3,2	0,00E+00	4
10853_at	YJR151C	DAN4	3,2	0,00E+00	5
9341_at	YMR291W	---	3,2	0,00E+00	5
4566_at	YHL042W	---	3,2	0,00E+00	2
10479_at	YKR098C	UBP11	3,2	0,00E+00	5
4528_i_at	YHR010W	RPL27A	3,2	1,12E-07	2
7989_at	YPL215W	CBP3	3,2	0,00E+00	3
10890_at	YJR142W	---	3,2	0,00E+00	3
6403_at	YDR079W	PET100	3,2	1,17E-12	5
9713_at	YML086C	ALO1	3,2	0,00E+00	4
5043_at	YGL019W	CKB1	3,2	0,00E+00	2
5788_at	YEL047C	---	3,2	0,00E+00	2
5620_at	YER091C	MET6	3,2	3,42E-08	2
7324_at	YBR056W	---	3,2	0,00E+00	5
6783_at	YCR088W	ABP1	3,2	0,00E+00	3
5298_at	YGL262W	---	3,2	0,00E+00	5
4449_at	YHR068W	DYS1	3,2	1,04E-07	2
4565_at	YHL043W	ECM34	3,1	0,00E+00	9
7258_at	YBR122C	MRPL36	3,1	0,00E+00	3
4448_at	YHR067W	HTD2	3,1	0,00E+00	4
4902_at	YGR112W	SHY1	3,1	0,00E+00	3
10416_at	YLL060C	GTT2	3,1	0,00E+00	5
10675_at	YKL109W	HAP4	3,1	0,00E+00	5
6425_at	YDR056C	---	3,1	0,00E+00	3
4820_at	YGR165W	MRPS35	3,1	0,00E+00	3
10301_at	YLR048W	RPS0B	3,1	2,48E-09	2
10160_at	YLR180W	SAM1	3,1	2,31E-12	2
8668_at	YOL120C	RPL18A	3,1	3,08E-09	2
5084_at	YGL068W	MNP1	3,1	0,00E+00	3
7190_i_at	YBR189W	RPS9B	3,1	9,15E-04	2
10296_at	YLR044C	PDC1	3,1	3,75E-08	2
5962_at	YDR493W	MZM1	3,1	0,00E+00	3
9044_at	YNL169C	PSD1	3,1	0,00E+00	2
9547_at	YMR105C	PGM2	3,1	0,00E+00	5
10295_at	YLR043C	TRX1	3,1	0,00E+00	2
4075_at	YIR039C	YPS6	3,1	0,00E+00	5
6492_at	YDL015C	TSC13	3,1	0,00E+00	4
4928_at	YGR092W	DBF2	3,1	0,00E+00	3
7037_s_at	YBR006W	UGA2	3,1	0,00E+00	5
7848_at	YPL087W	YDC1	3,1	0,00E+00	5
5573_at	YER133W	GLC7	3,1	5,92E-13	3
8929_at	YNL058C	---	3,1	0,00E+00	2
7736_at	YPR028W	YOP1	3,0	0,00E+00	4
5985_at	YDR471W	RPL27B	3,0	3,35E-08	2
4334_i_at	YHR174W	ENO2	3,0	2,40E-02	2
8298_at	YOR232W	MGE1	3,0	9,45E-11	3

8777_at	YNR065C	---	3,0	0,00E+00	4
10980_at	YJR051W	OSM1	3,0	5,92E-13	3
10414_at	YLL062C	MHT1	3,0	0,00E+00	3
4901_at	YGR111W	---	3,0	0,00E+00	5
7252_at	YBR116C	---	3,0	0,00E+00	5
5664_at	YER052C	HOM3	3,0	0,00E+00	2
11236_at	YJL190C	RPS22A	3,0	1,11E-06	2
6416_at	YDR047W	HEM12	3,0	0,00E+00	4
10770_at	YKL192C	ACP1	3,0	0,00E+00	3
5699_at	YER044C	ERG28	3,0	5,71E-12	4
10237_at	YLR118C	---	3,0	0,00E+00	4
10243_at	YLR079W	SIC1	3,0	0,00E+00	5
5785_at	YEL050C	RML2	3,0	0,00E+00	3
4173_at	YIL093C	RSM25	3,0	0,00E+00	3
4247_at	YIL154C	IMP2'	3,0	0,00E+00	5
9852_at	YLR448W	RPL6B	3,0	2,52E-08	2
10015_at	YLR300W	EXG1	3,0	4,67E-04	2
11387_at	YAL061W	BDH2	3,0	0,00E+00	5
5750_at	YEL040W	UTR2	3,0	5,92E-13	2
7152_at	YBR196C	PGI1	3,0	8,75E-10	2
9642_at	YMR020W	FMS1	3,0	0,00E+00	5
9513_at	YMR116C	ASC1	3,0	8,58E-11	2
8456_at	YOR119C	RIO1	3,0	5,15E-12	5
6299_at	YDR197W	CBS2	2,9	0,00E+00	3
9645_at	YML019W	OST6	2,9	0,00E+00	2
5698_at	YER043C	SAH1	2,9	2,14E-06	2
6447_at	YDR033W	MRH1	2,9	0,00E+00	3
10751_at	YKL168C	KKQ8	2,9	0,00E+00	5
10099_at	YLR251W	SYM1	2,9	0,00E+00	5
10270_at	YLR061W	RPL22A	2,9	1,03E-09	2
11290_at	YAR028W	---	2,9	1,74E-12	2
6248_at	YDR237W	MRPL7	2,9	0,00E+00	3
7116_at	YBR251W	MRPS5	2,9	0,00E+00	3
3940_at	YBR201W	DER1	2,9	0,00E+00	5
7829_at	YPL060W	LPE10	2,9	3,45E-12	5
5152_at	YGL137W	SEC27	2,9	0,00E+00	2
9621_at	YML002W	---	2,9	0,00E+00	5
11146_at	YJL101C	GSH1	2,9	0,00E+00	4
5123_at	YGL119W	ABC1	2,9	7,41E-12	4
3939_at	YBR006W	UGA2	2,9	0,00E+00	5
7835_at	YPL100W	ATG21	2,9	0,00E+00	5
8305_at	YOR239W	ABP140	2,9	0,00E+00	2
4999_at	YGR026W	---	2,9	2,27E-09	2
5571_at	YER131W	RPS26B	2,9	5,09E-06	2
8405_at	YOR158W	PET123	2,9	4,02E-12	5
4867_at	YGR122W	---	2,9	1,17E-12	5
6857_at	YCR024C	SLM5	2,9	1,74E-12	3
8814_at	YNR055C	HOL1	2,9	0,00E+00	4
11052_at	YJL016W	---	2,9	0,00E+00	5
10733_at	YKL140W	TGL1	2,8	0,00E+00	5
9159_at	YNL280C	ERG24	2,8	0,00E+00	2
4474_at	YHR045W	---	2,8	5,92E-13	2
11180_at	YJL158C	CIS3	2,8	1,54E-09	2
10643_at	YKL053C-A	MDM35	2,8	0,00E+00	3
4471_at	YHR042W	NCP1	2,8	0,00E+00	2
8840_at	YNR037C	RSM19	2,8	0,00E+00	3
6543_at	YDL055C	PSA1	2,8	6,28E-12	2
8531_at	YOR013W	IRC11	2,8	5,92E-13	2
4529_f_at	YHR010W	RPL27A	2,8	6,37E-09	2
4190_at	YIL074C	SER33	2,8	5,92E-13	2
8317_at	YOR250C	CLP1	2,8	0,00E+00	3
10893_s_at	YJR145C	RPS4A /// RPS4B	2,8	4,31E-05	2
10495_at	YKR070W	---	2,8	0,00E+00	3
9737_at	YML105C	SEC65	2,8	0,00E+00	2
6927_at	YCL049C	---	2,8	0,00E+00	3
10256_at	YLR092W	SUL2	2,8	0,00E+00	2
5030_at	YGL032C	AGA2	2,8	2,22E-05	2

11123_at	YJL079C	PRY1	2,8	2,52E-11	3
4417_at	YHR123W	EPT1	2,8	1,88E-09	2
10908_at	YJR115W	---	2,8	0,00E+00	5
10244_at	YLR080W	EMP46	2,8	0,00E+00	5
9120_at	YNL274C	GOR1	2,8	0,00E+00	5
6832_at	YCR044C	PER1	2,8	0,00E+00	2
5069_at	YGL082W	---	2,8	0,00E+00	3
9701_at	YML055W	SPC2	2,8	0,00E+00	2
11097_at	YJL060W	BNA3	2,8	0,00E+00	2
10569_at	YKR006C	MRPL13	2,8	0,00E+00	3
11079_at	YJL078C	PRY3	2,8	0,00E+00	4
5428_at	YFL052W	---	2,8	0,00E+00	5
7613_at	YPR127W	---	2,8	0,00E+00	5
4801_at	YGR191W	HIP1	2,8	0,00E+00	2
6188_at	YDR268W	MSW1	2,8	0,00E+00	3
8265_at	YOR289W	---	2,8	0,00E+00	5
10916_at	YJR123W	RPS5	2,8	1,23E-09	2
10242_at	YLR078C	BOS1	2,8	5,92E-13	3
5366_s_at	YFR024C-A	LSB3	2,8	0,00E+00	3
6424_at	YDR055W	PST1	2,8	0,00E+00	3
9448_at	YMR178W	---	2,8	0,00E+00	4
9512_at	YMR116C	ASC1	2,7	3,57E-09	2
6437_at	YDR022C	CIS1	2,7	5,92E-13	5
8671_at	YOL117W	RRI2	2,7	0,00E+00	5
6387_at	YDR107C	TMN2	2,7	1,27E-10	3
5403_at	YFL027C	GYP8	2,7	5,92E-13	3
4321_at	YHR208W	BAT1	2,7	8,88E-09	2
6240_at	YDR229W	IVY1	2,7	0,00E+00	5
4057_at	YIR021W	MRS1	2,7	0,00E+00	2
11388_at	YAL060W	BDH1	2,7	0,00E+00	5
8267_at	YOR291W	YPK9	2,7	0,00E+00	4
4397_at	YHR147C	MRPL6	2,7	0,00E+00	3
5552_at	YER158C	---	2,7	0,00E+00	5
8377_at	YOR175C	ALE1	2,7	0,00E+00	3
10232_at	YLR113W	HOG1	2,7	0,00E+00	4
4402_at	YHR109W	CTM1	2,7	5,92E-13	3
5957_at	YDR533C	HSP31	2,7	0,00E+00	4
6512_at	YDL040C	NAT1	2,7	0,00E+00	2
10769_at	YKL193C	SDS22	2,7	0,00E+00	5
6123_at	YDR337W	MRPS28	2,7	0,00E+00	3
7646_at	YPR114W	---	2,7	0,00E+00	2
10045_at	YLR286C	CTS1	2,7	0,00E+00	3
4535_at	YHR016C	YSC84	2,7	0,00E+00	5
10777_at	YKL186C	MTR2	2,7	5,92E-13	4
6909_at	YCL018W	LEU2	2,7	1,74E-08	2
6600_at	YDL130W	RPP1B	2,7	1,23E-08	2
7581_at	YPR183W	DPM1	2,7	0,00E+00	2
5307_at	YFR053C	HXK1	2,7	0,00E+00	5
8980_at	YNL097C	PHO23	2,6	0,00E+00	5
11371_at	YAL038W	CDC19	2,6	0,00E+00	2
6218_at	YDR253C	MET32	2,6	0,00E+00	4
10138_g_at	YLR202C	COQ9	2,6	0,00E+00	5
5714_at	YER014W	HEM14	2,6	0,00E+00	5
9327_at	YMR278W	PGM3	2,6	0,00E+00	5
5190_i_at	YGL189C	RPS26A	2,6	2,11E-06	2
6073_at	YDR376W	ARH1	2,6	0,00E+00	3
9726_at	YML073C	RPL6A	2,6	1,00E-07	2
8824_at	YNR021W	---	2,6	5,92E-13	2
6794_at	YCR099C	---	2,6	1,74E-12	5
6854_at	YCR021C	HSP30	2,6	0,00E+00	5
8788_at	YNR034w-a	---	2,6	0,00E+00	5
4967_at	YGR041W	BUD9	2,6	7,53E-08	2
7687_at	YPR067W	ISA2	2,6	0,00E+00	4
7191_f_at	YBR189W	RPS9B	2,6	1,79E-07	2
4971_at	YGR045C	---	2,6	3,46E-10	5
5363_at	YFR021W	ATG18	2,6	1,36E-11	4
9150_at	YNL289W	PCL1	2,6	5,00E-10	2

6036_at	YDR430C	CYM1	2,6	0,00E+00	3
10273_at	YLR064W	---	2,6	3,89E-10	4
7590_at	YPR147C	---	2,6	0,00E+00	3
9914_at	YLR423C	ATG17	2,6	0,00E+00	5
6432_at	YDR063W	AIM7	2,6	0,00E+00	4
8355_at	YOR198C	BFR1	2,6	1,47E-11	2
9443_at	YMR173W-A	---	2,6	0,00E+00	3
7565_at	YPR167C	MET16	2,6	5,92E-13	2
5009_at	YGL008C	PMA1	2,6	2,94E-07	2
6897_at	YCL035C	GRX1	2,6	0,00E+00	5
5673_at	YER058W	PET117	2,6	3,67E-10	3
6563_at	YDL079C	MRK1	2,6	0,00E+00	5
8870_at	YNL025C	SSN8	2,6	0,00E+00	5
5544_at	YER150W	SPI1	2,6	0,00E+00	5
5054_at	YGL053W	PRM8	2,6	0,00E+00	5
9761_at	YML124C	TUB3	2,6	0,00E+00	2
11325_at	YAR002C-A	ERP1	2,5	1,17E-12	2
5031_at	YGL031C	RPL24A	2,5	4,75E-07	2
8343_at	YOR187W	TUF1	2,5	0,00E+00	3
4779_f_at	YGR214W	RPS0A	2,5	2,06E-06	2
7857_at	YPL123C	RNY1	2,5	2,02E-06	5
10141_at	YLR203C	MSS51	2,5	0,00E+00	3
10018_at	YLR303W	MET17	2,5	3,19E-11	2
9534_at	YMR092C	AIP1	2,5	0,00E+00	3
11032_at	YJR010W	MET3	2,5	5,92E-13	2
7147_at	YBR191W	RPL21A	2,5	5,09E-06	2
7912_at	YPL158C	AIM44	2,5	0,00E+00	3
6409_at	YDR085C	AFR1	2,5	0,00E+00	5
9660_at	YML009C	MRPL39	2,5	0,00E+00	3
9391_at	YMR252C	---	2,5	0,00E+00	5
10136_at	YLR201C	COQ9	2,5	0,00E+00	5
8366_at	YOR165W	SEY1	2,5	3,45E-12	2
9634_at	YMR012W	CLU1	2,5	2,63E-11	2
8836_at	YNR033W	ABZ1	2,5	0,00E+00	5
11288_at	YAR023C	---	2,5	5,92E-13	5
4558_at	YHL004W	MRP4	2,5	9,10E-12	3
10069_f_at	YLR264W	RPS28B	2,5	1,24E-07	2
10087_at	YLR239C	LIP2	2,5	0,00E+00	3
5252_at	YGL259W	YPS5	2,5	0,00E+00	5
10174_at	YLR144C	ACF2	2,5	5,92E-13	4
10499_at	YKR074W	AIM29	2,5	0,00E+00	2
8896_at	YNL046W	---	2,5	0,00E+00	3
9483_at	YMR170C	ALD2	2,5	0,00E+00	5
10016_at	YLR301W	---	2,5	6,03E-11	2
6607_at	YDL124W	---	2,5	0,00E+00	5
7841_at	YPL094C	SEC62	2,5	1,02E-11	2
10350_at	YLR008C	PAM18	2,5	5,92E-13	2
9182_s_at	YNL302C	RPS19A /// RPS19B	2,5	0,00E+00	2
7617_at	YPR131C	NAT3	2,5	0,00E+00	2
10470_at	YKR091W	SRL3	2,5	2,80E-11	5
10076_at	YLR271W	---	2,5	5,92E-13	5
11106_at	YJL096W	MRPL49	2,5	0,00E+00	3
8323_at	YOR212W	STE4	2,5	0,00E+00	2
4547_at	YHL015W	RPS20	2,5	1,24E-10	2
8662_at	YOL127W	RPL25	2,5	1,66E-06	2
7186_at	YBR185C	MBA1	2,5	0,00E+00	3
4339_at	YHR179W	OYE2	2,5	0,00E+00	2
4788_at	YGR222W	PET54	2,5	0,00E+00	3
6569_at	YDL075W	RPL31A	2,5	2,96E-07	2
8717_at	YOL164W	BDS1	2,5	0,00E+00	3
8254_at	YOR278W	HEM4	2,5	0,00E+00	3
9887_at	YLR439W	MRPL4	2,4	0,00E+00	3
10654_at	YKL085W	MDH1	2,4	0,00E+00	3
7693_at	YPR073C	LTP1	2,4	1,31E-10	3
9651_at	YML013W	UBX2	2,4	0,00E+00	5
6364_at	YDR129C	SAC6	2,4	0,00E+00	5
6084_at	YDR387C	---	2,4	8,32E-10	3

10698_at	YKL130C	SHE2	2,4	0,00E+00	2
6541_at	YDL057W	---	2,4	0,00E+00	5
9183_s_at	YNL301C	RPL18A /// RPL18B	2,4	2,43E-08	2
4312_at	YHR198C	AIM18	2,4	0,00E+00	5
6216_at	YDR251W	PAM1	2,4	0,00E+00	3
11003_at	YJR025C	BNA1	2,4	0,00E+00	4
5076_f_at	YGL076C	RPL7A	2,4	8,97E-11	2
4164_at	YIL053W	RHR2	2,4	1,37E-07	2
5118_at	YGL124C	MON1	2,4	7,97E-12	5
5697_at	YER042W	MXR1	2,4	0,00E+00	3
5356_at	YFR014C	CMK1	2,4	1,25E-07	5
7160_at	YBR205W	KTR3	2,4	0,00E+00	2
8584_at	YOL023W	IFM1	2,4	5,94E-08	3
5647_s_at	YER074W	RPS24A /// RPS24B	2,4	1,54E-04	2
10278_at	YLR069C	MEF1	2,4	5,92E-13	3
7876_at	YPL104W	MSD1	2,4	0,00E+00	3
4315_at	YHR201C	PPX1	2,4	5,71E-11	2
10304_at	YLR050C	---	2,4	0,00E+00	2
9356_at	YMR262W	---	2,4	0,00E+00	5
10070_at	YLR265C	NEJ1	2,4	7,10E-09	4
11179_at	YJL159W	HSP150	2,4	0,00E+00	3
7767_at	YPL031C	PHO85	2,4	0,00E+00	5
9637_at	YMR015C	ERG5	2,4	1,18E-05	3
4729_i_at	YGR254W	ENO1	2,4	3,20E-06	9
6133_at	YDR347W	MRP1	2,4	0,00E+00	3
9444_at	YMR174C	PAI3	2,4	0,00E+00	5
8918_at	YNL069C	RPL16B	2,4	1,99E-05	2
4494_at	YHR021C	RPS27B	2,4	1,23E-06	2
7765_at	YPR013C	---	2,4	0,00E+00	5
8884_at	YNL011C	---	2,4	0,00E+00	3
9673_at	YML035C	AMD1	2,4	0,00E+00	4
4817_at	YGR207C	---	2,4	9,10E-12	2
8547_at	YOL015W	IRC10	2,4	1,52E-10	5
9454_at	YMR184W	ADD37	2,4	0,00E+00	2
5343_at	YFR044C	DUG1	2,4	0,00E+00	3
6559_f_at	YDL083C	RPS16B	2,4	1,97E-06	2
5317_s_at	YFL042C	---	2,4	0,00E+00	5
9556_at	YMR071C	TVP18	2,4	0,00E+00	3
11111_at	YJL091C	GWT1	2,4	0,00E+00	2
9338_at	YMR289W	ABZ2	2,4	5,87E-11	3
6304_at	YDR158W	HOM2	2,4	5,59E-08	2
7747_at	YPL006W	NCR1	2,4	0,00E+00	5
7844_at	YPL091W	GLR1	2,4	0,00E+00	3
5404_at	YFL026W	STE2	2,4	1,88E-09	2
9437_at	YMR210W	---	2,4	0,00E+00	5
6350_at	YDR115W	---	2,4	0,00E+00	3
8803_at	YNR045W	PET494	2,4	0,00E+00	5
10075_at	YLR270W	DCS1	2,4	0,00E+00	5
8411_at	YOR164C	GET4	2,4	1,23E-10	4
9942_at	YLR363C	NMD4	2,4	9,32E-10	4
5634_at	YER063W	THO1	2,4	0,00E+00	2
10993_at	YJR016C	ILV3	2,4	1,10E-06	2
6881_at	YCR003W	MRPL32	2,4	0,00E+00	3
5918_at	YDR539W	---	2,4	0,00E+00	5
8869_at	YNL026W	SAM50	2,4	0,00E+00	3
8269_f_at	YOR293W	RPS10A	2,4	4,56E-05	2
7104_at	YBR239C	ERT1	2,4	1,02E-11	4
6681_at	YDL183C	---	2,4	0,00E+00	5
7877_at	YPL103C	FMP30	2,4	4,02E-12	3
9574_at	YMR087W	---	2,4	0,00E+00	5
8623_at	YOL075C	---	2,4	1,49E-10	4
4826_at	YGR171C	MSM1	2,4	0,00E+00	3
4447_at	YHR066W	SSF1	2,4	2,17E-06	2
11213_at	YJL170C	ASG7	2,4	3,55E-07	5
4056_at	YIR020C	---	2,3	5,92E-13	5
11344_at	YAL023C	PMT2	2,3	0,00E+00	2
8948_at	YNL084C	END3	2,3	0,00E+00	5

11203_i_at	YJL177W	RPL17B	2,3	2,45E-04	2
6712_at	YDL241W	---	2,3	3,65E-08	2
6337_at	YDR146C	SWI5	2,3	1,17E-12	2
6723_at	YDL230W	PTP1	2,3	5,92E-13	3
10554_at	YKR037C	SPC34	2,3	0,00E+00	5
4074_at	YIR038C	GTT1	2,3	0,00E+00	5
6834_at	YCR046C	IMG1	2,3	0,00E+00	3
10448_s_at	YKL033W-A	---	2,3	0,00E+00	4
4353_at	YHR193C	EGD2	2,3	1,17E-12	2
8838_at	YNR035C	ARC35	2,3	0,00E+00	4
7328_at	YBR015C	MNN2	2,3	0,00E+00	3
7845_s_at	YPL090C	RPS6A /// RPS6B	2,3	5,81E-06	2
11059_at	YJL053W	PEP8	2,3	5,15E-12	3
7058_at	YBR285W	---	2,3	0,00E+00	5
5000_i_at	YGR027C	RPS25A	2,3	1,45E-02	2
10922_at	YJR083C	ACF4	2,3	1,17E-12	5
4354_at	YHR194W	MDM31	2,3	0,00E+00	3
6141_at	YDR311W	TFB1	2,3	0,00E+00	2
5678_at	YER023W	PRO3	2,3	7,41E-12	2
8914_at	YNL073W	MSK1	2,3	0,00E+00	5
8718_at	YOL163W	---	2,3	1,21E-10	4
7803_at	YPL040C	ISM1	2,3	0,00E+00	3
8235_at	YOR301W	RAX1	2,3	0,00E+00	2
6104_at	YDR363W	ESC2	2,3	2,90E-10	5
7826_at	YPL063W	TIM50	2,3	1,52E-11	4
11041_at	YJL026W	RNR2	2,3	0,00E+00	2
10324_at	YLR028C	ADE16	2,3	0,00E+00	3
7162_at	YBR206W	---	2,3	7,26E-09	2
10334_at	YLL009C	COX17	2,3	0,00E+00	3
10954_at	YJR070C	LIA1	2,3	2,08E-11	2
7969_at	YPL191C	---	2,3	3,90E-08	5
6566_at	YDL077C	VAM6	2,3	0,00E+00	3
6072_at	YDR375C	BCS1	2,3	0,00E+00	3
8554_at	YOL008W	COQ10	2,3	2,88E-12	4
4860_at	YGR159C	NSR1	2,3	3,92E-04	2
7275_at	YBR094W	PBY1	2,3	2,28E-08	3
7322_at	YBR054W	YRO2	2,3	0,00E+00	5
4887_at	YGR141W	VPS62	2,3	0,00E+00	5
10049_at	YLR289W	GUF1	2,3	1,17E-10	3
8316_at	YOR249C	APC5	2,3	4,58E-12	3
11206_f_at	YJL177W	RPL17B	2,3	3,50E-07	2
7038_s_at	YBR201W	DER1	2,3	1,04E-09	2
4525_at	YHR007C	ERG11	2,3	3,51E-04	2
7943_at	YPL172C	COX10	2,3	0,00E+00	3
6365_at	YDR130C	FIN1	2,3	4,02E-12	3
9295_g_at	YMR122w-a	---	2,3	0,00E+00	2
4883_at	YGR137W	---	2,3	4,35E-10	5
6278_at	YDR177W	UBC1	2,3	0,00E+00	4
10979_at	YJR050W	ISY1	2,3	0,00E+00	5
7908_at	YPL162C	---	2,3	3,13E-10	3
5088_at	YGL064C	MRH4	2,3	5,92E-13	3
4357_at	YHR152W	SPO12	2,3	1,74E-12	3
6107_at	YDR365C	ESF1	2,3	2,90E-08	2
9684_s_at	YML026C	RPS18A /// RPS18B	2,3	1,36E-11	2
10756_at	YKL163W	PIR3	2,3	3,47E-04	5
4818_at	YGR208W	SER2	2,3	1,53E-05	2
4532_at	YHR013C	ARD1	2,3	7,85E-10	2
7150_at	YBR194W	AIM4	2,3	0,00E+00	3
9772_at	YML115C	VAN1	2,3	0,00E+00	2
10228_at	YLR109W	AHP1	2,3	0,00E+00	4
5100_at	YGL097W	SRM1	2,3	5,92E-13	2
7942_at	YPL173W	MRPL40	2,3	0,00E+00	3
4799_at	YGR189C	CRH1	2,3	1,40E-09	2
6902_at	YCL030C	HIS4	2,3	1,75E-11	2
7795_at	YPL048W	CAM1	2,3	0,00E+00	3
9635_at	YMR013C	SEC59	2,3	6,88E-07	2
5670_i_at	YER056C-A	RPL34A /// RPL34B	2,3	4,73E-02	2

5937_at	YDR513W	GRX2	2,2	0,00E+00	5
10964_at	YJR080C	AIM24	2,2	3,22E-10	3
9423_at	YMR196W	---	2,2	0,00E+00	5
7179_at	YBR177C	EHT1	2,2	0,00E+00	3
10147_at	YLR167W	RPS31	2,2	0,00E+00	2
8001_at	YPL249C	GYP5	2,2	1,25E-11	3
7448_at	YBL092W	RPL32	2,2	2,22E-06	2
10120_at	YLR228C	ECM22	2,2	3,89E-10	5
4813_at	YGR203W	YCH1	2,2	0,00E+00	2
7228_at	YBR137W	---	2,2	0,00E+00	5
8478_at	YOR096W	RPS7A	2,2	3,14E-07	2
10327_at	YLR030W	---	2,2	4,73E-11	5
5744_at	YEL002C	WBP1	2,2	0,00E+00	2
6710_at	YDL243C	AAD4	2,2	4,11E-08	5
9975_at	YLR351C	NIT3	2,2	0,00E+00	2
11058_at	YJL054W	TIM54	2,2	1,60E-07	2
8837_at	YNR034W	SOL1	2,2	1,86E-11	5
6561_at	YDL081C	RPP1A	2,2	9,66E-09	2
8953_at	YNL079C	TPM1	2,2	4,07E-11	4
10254_at	YLR090W	XDJ1	2,2	0,00E+00	5
4238_at	YIL164C	NIT1	2,2	5,92E-13	4
8899_at	YNL042W	BOP3	2,2	0,00E+00	4
10302_g_at	YLR048W	RPS0A /// RPS0B	2,2	1,38E-06	2
6572_at	YDL072C	YET3	2,2	0,00E+00	5
7241_at	YBR105C	VID24	2,2	5,92E-13	5
8885_at	YNL010W	---	2,2	2,60E-10	2
9294_at	YMR122w-a	---	2,2	0,00E+00	2
7862_at	YPL118W	MRP51	2,2	9,10E-12	3
5227_at	YGL198W	YIP4	2,2	0,00E+00	4
5652_at	YER079W	---	2,2	0,00E+00	5
6692_at	YDL216C	RRI1	2,2	0,00E+00	5
7657_at	YPR081C	GRS2	2,2	4,51E-11	5
7811_at	YPL079W	RPL21B	2,2	3,52E-06	2
10021_at	YLR306W	UBC12	2,2	0,00E+00	4
9496_at	YMR142C	RPL13B	2,2	4,21E-07	2
4172_at	YIL094C	LYS12	2,2	1,61E-07	2
6134_at	YDR348C	---	2,2	1,17E-12	2
8036_at	YPL259C	APM1	2,2	1,79E-09	4
10895_at	YJR147W	HMS2	2,2	5,92E-13	2
4149_at	YIL070C	MAM33	2,2	5,92E-13	3
6899_at	YCL033C	MXR2	2,2	0,00E+00	5
9045_at	YNL168C	FMP41	2,2	0,00E+00	3
4503_at	YHR029C	YHI9	2,2	5,92E-13	2
5146_at	YGL143C	MRF1	2,2	9,25E-04	3
8900_at	YNL041C	COG6	2,2	0,00E+00	2
7265_at	YBR087W	RFC5	2,2	3,67E-09	2
11205_i_at	YJL177W	RPL17B	2,2	1,13E-03	2
4115_at	YIL010W	DOT5	2,2	0,00E+00	3
4824_at	YGR169C	PUS6	2,2	2,88E-12	2
8922_at	YNL066W	SUN4	2,2	0,00E+00	2
5219_at	YGL206C	CHC1	2,2	0,00E+00	5
4786_at	YGR220C	MRPL9	2,2	0,00E+00	3
10100_at	YLR252W	---	2,2	0,00E+00	5
5225_at	YGL200C	EMP24	2,2	0,00E+00	2
6795_at	YCR100C	---	2,2	0,00E+00	5
9323_at	YMR315W	---	2,2	0,00E+00	3
6785_at	YCR090C	---	2,2	5,00E-09	2
9890_s_at	YLR441C	RPS1A /// RPS1B	2,2	6,55E-05	2
5682_at	YER027C	GAL83	2,2	0,00E+00	4
8591_at	YOL016C	CMK2	2,2	1,31E-09	5
9478_at	YMR166C	---	2,2	0,00E+00	3
5707_at	YER007C-A	TMA20	2,2	0,00E+00	2
5745_at	YEL001C	IRC22	2,2	1,03E-10	2
10283_at	YLR031W	---	2,2	7,13E-10	5
11181_at	YJL157C	FAR1	2,2	4,67E-03	2
6716_at	YDL237W	AIM6	2,2	0,00E+00	3
8612_at	YOL040C	RPS15	2,2	1,52E-11	2

10778_at	YKL185W	ASH1	2,2	0,00E+00	3
9714_at	YML085C	TUB1	2,2	0,00E+00	2
7034_i_at	YBR126w-a	---	2,2	0,00E+00	5
10986_at	YJR057W	CDC8	2,2	1,24E-07	3
11105_at	YJL097W	PHS1	2,2	0,00E+00	2
10073_at	YLR268W	SEC22	2,2	5,92E-13	3
9948_f_at	YLR367W	RPS22B	2,2	9,23E-09	2
8368_i_at	YOR167C	RPS28A	2,1	8,86E-03	2
7882_f_at	YPL143W	RPL33A	2,1	3,65E-06	2
5988_at	YDR474C	JIP4	2,1	0,00E+00	3
6612_at	YDL119C	---	2,1	1,36E-11	4
8300_f_at	YOR234C	RPL33B	2,1	6,40E-07	2
10132_at	YLR197W	NOP56	2,1	4,46E-08	2
6419_at	YDR050C	TPI1	2,1	1,30E-07	2
6609_at	YDL122W	UBP1	2,1	1,34E-09	2
4737_at	YGR261C	APL6	2,1	0,00E+00	2
4458_at	YHR078W	---	2,1	0,00E+00	5
4072_at	YIR036C	IRC24	2,1	0,00E+00	5
7218_at	YBR127C	VMA2	2,1	0,00E+00	2
5954_at	YDR530C	APA2	2,1	0,00E+00	5
7072_s_at	YBR299W	MAL12 /// MAL32	2,1	0,00E+00	5
4980_at	YGR008C	STF2	2,1	0,00E+00	5
9735_at	YML107C	PML39	2,1	6,20E-11	3
5237_at	YGL232W	TAN1	2,1	5,93E-11	2
5329_s_at	YFR031C-A	RPL2A /// RPL2B	2,1	2,34E-03	2
7606_at	YPR162C	ORC4	2,1	0,00E+00	2
9897_f_at	YLR406C	RPL31B	2,1	8,31E-11	2
9086_at	YNL217W	---	2,1	2,08E-11	2
4990_at	YGR017W	---	2,1	5,92E-13	2
11339_at	YAL028W	FRT2	2,1	0,00E+00	5
8936_at	YNL096C	RPS7B	2,1	7,99E-11	2
6564_at	YDL079C	MRK1	2,1	5,21E-10	5
7840_at	YPL095C	EEB1	2,1	9,18E-07	4
6099_at	YDR358W	GGA1	2,1	0,00E+00	5
5005_at	YGL012W	ERG4	2,1	6,63E-08	2
6426_at	YDR057W	YOS9	2,1	4,07E-09	3
4730_s_at	YGR254W	ENO1 /// ENO2	2,1	2,30E-06	2
5779_at	YEL056W	HAT2	2,1	2,42E-08	3
10800_at	YKL212W	SAC1	2,1	1,03E-08	2
10736_at	YKL137W	CMC1	2,1	1,24E-10	3
8724_at	YOL155C	HPF1	2,1	0,00E+00	5
9430_at	YMR203W	TOM40	2,1	5,71E-12	3
11204_s_at	YJL177W	RPL17A /// RPL17B	2,1	3,25E-07	2
4574_at	YHL034C	SBP1	2,1	0,00E+00	3
8603_at	YOL049W	GSH2	2,1	7,96E-06	2
5191_f_at	YGL189C	RPS26A	2,1	7,57E-08	2
6599_at	YDL130W	RPP1B	2,1	2,79E-05	2
5746_at	YER001W	MNN1	2,1	2,50E-03	2
8917_at	YNL070W	TOM7	2,1	1,19E-10	4
10557_f_at	YKL006W	RPL14A	2,1	7,82E-07	2
10494_at	YKR069W	MET1	2,1	1,17E-12	2
10738_i_at	YKL180W	RPL17A	2,1	3,60E-04	2
8613_at	YOL039W	RPP2A	2,1	1,35E-06	2
5174_at	YGL161C	YIP5	2,1	0,00E+00	3
7219_at	YBR128C	ATG14	2,1	1,91E-11	5
7316_s_at	YBR048W	RPS11A /// RPS11B	2,1	3,01E-07	2
6850_at	YCR018C	SRD1	2,1	1,05E-09	2
4514_at	YHR039C-B	VMA10	2,1	1,17E-12	2
6546_at	YDL052C	SLC1	2,1	0,00E+00	2
8598_at	YOL053W	AIM39	2,1	2,29E-10	3
8375_at	YOR173W	DCS2	2,1	0,00E+00	5
5127_at	YGL115W	SNF4	2,1	0,00E+00	3
4517_at	YHR001W	OSH7	2,1	0,00E+00	3
8981_at	YNL096C	RPS7B	2,1	2,29E-07	2
9869_at	YLR264c-a	---	2,1	1,15E-08	2
7638_at	YPR107C	YTH1	2,1	2,18E-10	3
11158_i_at	YJL136C	RPS21B	2,1	5,22E-07	2

8166_at	YOR367W	SCP1	2,1	1,17E-12	3
5943_at	YDR519W	FPR2	2,1	1,43E-06	2
9551_at	YMR109W	MYO5	2,1	5,92E-13	4
6641_at	YDL179W	PCL9	2,1	1,04E-09	3
8296_at	YOR275C	RIM20	2,1	4,43E-09	3
10166_at	YLR185W	RPL37A	2,1	1,29E-06	2
9505_at	YMR150C	IMP1	2,1	0,00E+00	3
10053_at	YLR293C	GSP1	2,1	0,00E+00	2
10385_i_at	YLL045C	RPL8A /// RPL8B	2,1	2,23E-02	2
6630_at	YDL145C	COP1	2,1	0,00E+00	2
10096_at	YLR248W	RCK2	2,1	0,00E+00	5
10799_at	YKL213C	DOA1	2,1	2,88E-12	3
10386_s_at	YLL045C	RPL8A /// RPL8B	2,1	1,41E-05	2
10629_at	YKL067W	YNK1	2,1	0,00E+00	5
4117_at	YIL009C-A	EST3	2,1	7,28E-11	2
7217_at	YBR126C	TPS1	2,1	0,00E+00	5
5763_at	YEL027W	CUP5	2,1	0,00E+00	2
6583_at	YDL103C	QRI1	2,1	2,37E-09	2
8632_at	YOL065C	INP54	2,1	1,17E-12	5
3851_s_at	YJR161C	COS5 /// COS7	2,1	0,00E+00	5
11126_at	YJL123C	MTC1	2,1	5,92E-13	3
8825_at	YNR022C	MRPL50	2,1	9,23E-09	3
8813_at	YNR054C	ESF2	2,1	1,88E-06	2
4244_at	YIL157C	COA1	2,1	1,97E-11	5
6445_at	YDR031W	MIC14	2,1	4,07E-11	5
4498_at	YHR024C	MAS2	2,1	1,50E-10	3
9330_at	YMR281W	GPI12	2,1	7,37E-06	5
5536_at	YER187W	---	2,1	0,00E+00	5
9590_at	YMR060C	SAM37	2,1	0,00E+00	3
10754_at	YKL165C	MCD4	2,1	2,44E-06	2
10739_f_at	YKL180W	RPL17A	2,1	9,66E-09	2
8304_at	YOR238W	---	2,1	0,00E+00	4
4949_at	YGR068C	ART5	2,1	2,30E-07	3
8176_at	YOR377W	ATF1	2,1	6,36E-11	5
11164_at	YJL131C	AIM23	2,1	6,84E-12	3
9033_at	YNL181W	---	2,1	8,59E-08	3
6320_at	YDR174W	HMO1	2,1	0,00E+00	4
7621_at	YPR133W-A	TOM5	2,0	1,52E-11	2
8260_at	YOR284W	HUA2	2,0	2,27E-09	3
10142_at	YLR204W	QRI5	2,0	2,93E-08	3
7412_at	YBL040C	ERD2	2,0	3,61E-10	2
9717_at	YML082W	---	2,0	4,58E-12	2
3738_s_at	YML132W	COS1 /// COS2 /// COS	2,0	0,00E+00	5
7207_at	YBR161W	CSH1	2,0	5,17E-11	4
4809_at	YGR199W	PMT6	2,0	4,58E-12	2
4941_at	YGR060W	ERG25	2,0	1,00E-03	2
6446_at	YDR032C	PST2	2,0	0,00E+00	5
5312_s_at	YFL043C	---	2,0	0,00E+00	5
4343_at	YHR183W	GND1	2,0	3,77E-09	2
4774_at	YGR209C	TRX2	2,0	0,00E+00	5
10123_at	YLR231C	BNA5	2,0	2,07E-09	3
6021_f_at	YDR461W	MFA1	2,0	1,52E-03	2
7633_s_at	YPR102C	RPL11A /// RPL11B	2,0	3,04E-05	2
4487_at	YHR059W	FYV4	2,0	1,11E-05	3
10307_at	YLR011W	LOT6	2,0	2,15E-06	3
9704_at	YML052W	SUR7	2,0	1,17E-12	2
6489_at	YDL018C	ERP3	2,0	1,41E-09	5
4739_at	YGR263C	SAY1	2,0	3,79E-06	3
5667_at	YER055C	HIS1	2,0	1,07E-09	2
7618_f_at	YPR132W	RPS23B	2,0	1,92E-04	2
5322_at	YFR025C	HIS2	2,0	0,00E+00	2
9983_at	YLR359W	ADE13	2,0	5,71E-12	2
5724_at	YEL021W	URA3	2,0	5,92E-13	2
5633_at	YER062C	HOR2	2,0	2,33E-08	2
5651_at	YER078C	ICP55	2,0	7,61E-10	3
8252_at	YOR276W	CAF20	2,0	1,51E-09	2
10474_s_at	YKR094C	RPL40A /// RPL40B	2,0	5,57E-09	2

10536_at	YKR019C	IRS4	2,0	5,92E-13	5
7413_at	YBL039C	URA7	2,0	2,92E-06	2
6066_at	YDR415C	---	2,0	1,30E-11	2
10515_at	YKR043C	---	2,0	6,36E-11	2
7564_at	YPR166C	MRP2	2,0	1,08E-10	3
5244_at	YGL226W	MTC3	2,0	0,00E+00	3
9864_at	YLR460C	---	2,0	5,92E-13	2
6368_at	YDR089W	---	2,0	2,62E-10	4
4116_at	YIL009C-A	EST3	2,0	5,92E-13	2
5644_at	YER071C	---	2,0	3,45E-12	3
10803_at	YKL209C	STE6	2,0	3,14E-10	2
9896_f_at	YLR406C	RPL31B	2,0	8,69E-10	2
5327_at	YFR030W	MET10	2,0	3,47E-05	2
9376_at	YMR237W	BCH1	2,0	0,00E+00	3
10391_at	YLL040C	VPS13	2,0	5,76E-10	5
8334_at	YOR222W	ODC2	2,0	5,92E-13	2
9336_at	YMR287C	DSS1	2,0	1,08E-03	4
10760_at	YKL206C	ADD66	2,0	5,92E-13	3
4423_at	YHR087W	RTC3	2,0	5,92E-13	5
8984_at	YNL138W	SRV2	2,0	0,00E+00	3
4899_at	YGR109C	CLB6	2,0	1,34E-05	2
6579_at	YDL107W	MSS2	2,0	1,51E-09	5
4033_f_at	YIR044C	COS7	2,0	1,63E-10	5
5352_at	YFR010W	UBP6	2,0	1,74E-12	3
8417_at	YOR125C	CAT5	2,0	5,92E-13	5
8491_at	YOR063W	RPL3	2,0	3,77E-09	2
8699_at	YOL135C	MED7	2,0	4,33E-10	2
9137_at	YNL257C	SIP3	2,0	9,55E-07	4
9572_at	YMR086W	---	2,0	7,84E-09	5
9685_at	YML025C	YML6	2,0	4,02E-11	3
8848_at	YNL001W	DOM34	2,0	3,32E-07	2
8380_at	YOR178C	GAC1	2,0	0,00E+00	5
10905_at	YJR112W	NNF1	2,0	5,53E-05	2
10253_at	YLR089C	ALT1	2,0	0,00E+00	3
9909_at	YLR418C	CDC73	2,0	6,15E-09	4
6080_at	YDR382W	RPP2B	2,0	0,00E+00	2
5247_at	YGL223C	COG1	2,0	1,00E-07	3
4434_at	YHR097C	---	2,0	0,00E+00	5
4203_at	YIL108W	---	2,0	0,00E+00	5
5119_at	YGL123W	RPS2	2,0	4,38E-07	2
11069_at	YJL044C	GYP6	2,0	7,53E-10	5
10648_at	YKL091C	---	2,0	0,00E+00	5
9924_at	YLR389C	STE23	2,0	6,80E-04	2
10129_at	YLR194C	---	2,0	4,43E-09	2
11234_at	YJL192C	SOP4	2,0	5,14E-09	2
5260_at	YGL253W	HXK2	2,0	1,39E-04	2
8274_at	YOR253W	NAT5	2,0	6,47E-11	2
5060_at	YGL047W	ALG13	2,0	0,00E+00	3
4909_f_at	YGR118W	RPS23A	2,0	2,26E-05	2
8829_at	YNR026C	SEC12	2,0	1,30E-11	2
3852_f_at	YJR161C	COS5 /// COS7	2,0	4,73E-11	5
4769_at	YGR249W	MGA1	2,0	4,44E-08	5
5087_at	YGL065C	ALG2	2,0	4,58E-12	2
6430_at	YDR061W	---	2,0	0,00E+00	3
4918_at	YGR082W	TOM20	2,0	0,00E+00	2
4898_at	YGR108W	CLB1	2,0	1,26E-03	2
5588_at	YER103W	SSA4	2,0	0,00E+00	5
7161_g_at	YBR205W	KTR3	2,0	1,74E-12	2
3947_at	YKL033W-A	---	2,0	0,00E+00	4
5349_at	YFR007W	YFH7	2,0	1,44E-10	3
8916_at	YNL071W	LAT1	2,0	1,12E-09	5
7589_at	YPR146C	---	2,0	6,86E-10	3
6551_at	YDL090C	RAM1	2,0	1,68E-09	5
5672_at	YER057C	HMF1	2,0	5,83E-10	2
7425_s_at	YBL072C	RPS8A /// RPS8B	2,0	6,12E-05	2
8202_at	YOR358W	HAP5	2,0	0,00E+00	5
9477_at	YMR165C	PAH1	2,0	7,39E-11	5

5011_at	YGL006W	PMC1	2,0	0,00E+00	5
6147_at	YDR316W	OMS1	2,0	0,00E+00	3
8370_at	YOR168W	GLN4	2,0	8,48E-07	2
10159_at	YLR179C	---	2,0	4,29E-11	2
8704_at	YOL130W	ALR1	2,0	0,00E+00	5
7570_at	YPR172W	---	2,0	5,92E-13	5
10674_at	YKL110C	KTI12	2,0	5,92E-13	2
7692_at	YPR072W	NOT5	2,0	1,80E-09	4
10661_at	YKL079W	SMY1	2,0	3,96E-11	4
7628_at	YPR140W	TAZ1	2,0	2,88E-12	3
9354_at	YMR260C	TIF11	2,0	1,59E-04	2
10885_at	YJR137C	MET5	2,0	3,42E-06	2
7683_at	YPR063C	---	2,0	2,88E-12	2

Genes higher expressed in aerobic SP

Probeset id	Systematic name	Standard name	Fold change	Adjusted P	Cluster
8020_s_at	YPL276W	FDH1	484,7	0,00E+00	1
8142_s_at	YOR388C	FDH1 /// FDH2	409,9	0,00E+00	1
9309_at	YMR303C	ADH2	296,8	0,00E+00	8
6221_at	YDR256C	CTA1	246,9	0,00E+00	1
8192_at	YOR348C	PUT4	206,2	0,00E+00	1
6887_at	YCR010C	ADY2	138,6	0,00E+00	1
11297_at	YAR035W	YAT1	111,2	0,00E+00	8
9958_at	YLR377C	FBP1	95,7	0,00E+00	1
10934_at	YJR095W	SFC1	90,5	0,00E+00	1
5220_at	YGL205W	POX1	75,4	0,00E+00	1
6082_at	YDR384C	ATO3	64,8	0,00E+00	8
8959_at	YNL117W	MLS1	63,9	0,00E+00	1
7753_at	YPR001W	CIT3	60,4	0,00E+00	1
7594_at	YPR151C	SUE1	55,1	0,00E+00	1
5636_at	YER065C	ICL1	53,3	0,00E+00	8
10795_at	YKL217W	JEN1	48,7	0,00E+00	1
5679_at	YER024W	YAT2	43,6	0,00E+00	1
11113_at	YJL089W	SIP4	39,7	0,00E+00	1
10043_at	YLR284C	ECI1	34,9	0,00E+00	1
9640_at	YMR018W	---	32,9	0,00E+00	1
5738_at	YEL008W	---	32,5	0,00E+00	1
7409_at	YBL043W	ECM13	30,5	0,00E+00	1
10478_at	YKR097W	PCK1	28,5	0,00E+00	1
10143_at	YLR205C	HMX1	25,6	0,00E+00	1
7754_at	YPR002W	PDH1	22,1	0,00E+00	8
5715_at	YER015W	FAA2	21,5	0,00E+00	1
8663_at	YOL126C	MDH2	20,9	0,00E+00	1
5915_at	YDR536W	STL1	20,5	0,00E+00	1
8437_at	YOR100C	CRC1	19,9	0,00E+00	1
11356_at	YAL054C	ACS1	18,9	0,00E+00	1
10154_at	YLR174W	IDP2	18,8	0,00E+00	1
4584_at	YHL024W	RIM4	17,5	0,00E+00	1
4432_at	YHR096C	HXT5	16,7	0,00E+00	1
10996_at	YJR019C	TES1	15,8	0,00E+00	1
7422_at	YBL075C	SSA3	15,5	0,00E+00	1
11114_at	YJL088W	ARG3	15,4	0,00E+00	7
8886_at	YNL009W	IDP3	15,1	0,00E+00	1
8181_at	YOR382W	FIT2	14,2	0,00E+00	1
7981_at	YPL223C	GRE1	14,1	0,00E+00	1
8465_at	YOR084W	LPX1	14,1	0,00E+00	1
9057_at	YNL202W	SPS19	13,7	0,00E+00	1
10572_at	YKR009C	FOX2	13,7	0,00E+00	1
9702_at	YML054C	CYB2	13,2	0,00E+00	1
8444_at	YOR107W	RGS2	12,4	0,00E+00	1
7923_at	YPL147W	PXA1	12,2	0,00E+00	1
4948_at	YGR067C	---	12,2	0,00E+00	1
9756_at	YML089C	---	12,1	0,00E+00	1
9098_at	YNL251C	NRD1	11,9	0,00E+00	7
5977_at	YDR508C	GNP1	11,8	0,00E+00	7
7867_at	YPL113C	---	11,6	0,00E+00	1
9111_at	YNL237W	YTP1	11,4	0,00E+00	1
7758_at	YPR006C	ICL2	11,1	0,00E+00	8
10172_at	YLR142W	PUT1	10,8	0,00E+00	1
8382_at	YOR180C	DCI1	10,7	0,00E+00	8
4365_at	YHR160C	PEX18	10,7	0,00E+00	1
6077_at	YDR380W	ARO10	10,7	0,00E+00	1
10977_at	YJR048W	CYC1	10,6	0,00E+00	6
10072_at	YLR267W	BOP2	10,2	0,00E+00	1
4241_at	YIL160C	POT1	10,1	0,00E+00	1
6081_at	YDR383C	NKP1	9,5	0,00E+00	7
8887_at	YNL008C	ASI3	9,2	0,00E+00	7

10511_at	YKR039W	GAP1	9,1	0,00E+00	1
4900_at	YGR110W	CLD1	9,0	0,00E+00	1
8687_at	YOL147C	PEX11	8,9	0,00E+00	8
9614_at	YMR036C	MIH1	8,8	0,00E+00	1
10775_at	YKL188C	PXA2	8,2	0,00E+00	1
8997_at	YNL125C	ESBP6	8,1	0,00E+00	1
10472_at	YKR093W	PTR2	7,9	0,00E+00	8
4386_at	YHR137W	ARO9	7,5	0,00E+00	1
5406_at	YFL024C	EPL1	7,5	0,00E+00	7
7810_at	YPL033C	SRL4	7,5	0,00E+00	1
9724_at	YML075C	HMG1	7,3	0,00E+00	8
7853_at	YPL082C	MOT1	7,2	0,00E+00	7
3988_at	Q0115	COX3	7,1	0,00E+00	7
10023_at	YLR308W	CDA2	6,8	0,00E+00	1
7957_at	YPL201C	YIG1	6,8	0,00E+00	1
5612_at	YER084W	---	6,8	0,00E+00	1
9064_at	YNL195C	---	6,8	0,00E+00	1
6884_at	YCR006C	---	6,7	0,00E+00	1
9500_at	YMR145C	NDE1	6,6	0,00E+00	1
4362_at	YHR157W	REC104	6,6	4,77E-09	1
11185_at	YJL153C	INO1	6,5	0,00E+00	8
5769_at	YEL065W	SIT1	6,4	0,00E+00	8
7685_at	YPR065W	ROX1	6,4	0,00E+00	1
8199_at	YOR355W	GDS1	6,3	0,00E+00	7
10027_at	YLR312C	---	6,2	0,00E+00	1
7580_at	YPR182W	SMX3	6,2	0,00E+00	7
8666_at	YOL123W	HRP1	6,1	0,00E+00	1
4209_at	YIL101C	XBP1	5,9	0,00E+00	1
5007_at	YGL010W	---	5,9	0,00E+00	8
9624_at	YMR002W	MIC17	5,8	0,00E+00	8
8236_at	YOR302W	---	5,8	0,00E+00	7
8162_at	YOR363C	PIP2	5,8	0,00E+00	1
8867_at	YNR019W	ARE2	5,8	0,00E+00	1
9416_at	YMR191W	SPG5	5,7	0,00E+00	1
6346_at	YDR111C	ALT2	5,7	0,00E+00	1
4248_at	YIL153W	RRD1	5,7	0,00E+00	7
9612_at	YMR034C	---	5,7	0,00E+00	1
8850_at	YNR002C	ATO2	5,7	0,00E+00	1
7602_at	YPR158W	CUR1	5,7	0,00E+00	7
7681_at	YPR061C	JID1	5,7	0,00E+00	1
5585_at	YER145C	FTR1	5,6	0,00E+00	6
6458_at	YDR043C	NRG1	5,5	0,00E+00	1
8217_at	YOR327C	SNC2	5,5	0,00E+00	7
8679_at	YOL109W	ZEO1	5,5	0,00E+00	7
6078_at	YDR381W	YRA1	5,4	0,00E+00	7
5553_at	YER159C	BUR6	5,4	0,00E+00	1
10518_at	YKR046C	PET10	5,4	0,00E+00	1
6556_at	YDL085W	NDE2	5,4	0,00E+00	1
9934_at	YLR399C	BDF1	5,4	0,00E+00	7
6079_at	YDR381W	YRA1	5,4	0,00E+00	7
6094_at	YDR397C	NCB2	5,4	0,00E+00	7
9026_at	YNL142W	MEP2	5,3	0,00E+00	8
8182_at	YOR383C	FIT3	5,3	0,00E+00	1
7076_at	YBR056w-a	---	5,3	0,00E+00	8
7140_at	YBR230C	OM14	5,2	0,00E+00	1
11102_at	YJL100W	LSB6	5,2	0,00E+00	8
10592_at	YKL015W	PUT3	5,2	0,00E+00	7
8207_at	YOR317W	FAA1	5,1	0,00E+00	1
8881_at	YNL014W	HEF3	5,1	0,00E+00	1
4390_at	YHR140W	---	5,1	0,00E+00	1
6487_at	YDL020C	RPN4	5,0	0,00E+00	1
9588_at	YMR058W	FET3	5,0	0,00E+00	6
7873_at	YPL107W	---	5,0	0,00E+00	8
9386_at	YMR246W	FAA4	4,9	0,00E+00	7
7311_at	YBR043C	QDR3	4,9	0,00E+00	1
4097_at	YIR016W	---	4,9	0,00E+00	1
8571_at	YOL036W	---	4,9	0,00E+00	1

7069_at	YBR296C	PHO89	4,9	0,00E+00	1
9898_at	YLR407W	---	4,8	0,00E+00	7
8664_at	YOL125W	TRM13	4,8	0,00E+00	8
9490_at	YMR136W	GAT2	4,8	0,00E+00	7
7457_at	YBL084C	CDC27	4,8	0,00E+00	8
5181_at	YGL154C	LYS5	4,7	0,00E+00	1
5606_at	YER121W	---	4,7	0,00E+00	1
8686_at	YOL148C	SPT20	4,7	0,00E+00	7
8556_at	YOL006C	TOP1	4,6	0,00E+00	7
5690_at	YER035W	EDC2	4,6	0,00E+00	1
7914_at	YPL156C	PRM4	4,5	0,00E+00	1
7445_at	YBL095W	---	4,5	0,00E+00	8
4212_g_at	YIL099W	SGA1	4,5	0,00E+00	1
7280_at	YBR099C	---	4,5	0,00E+00	1
11068_at	YJL045W	---	4,5	0,00E+00	1
8294_at	YOR273C	TPO4	4,4	0,00E+00	1
8214_at	YOR324C	FRT1	4,4	0,00E+00	8
5051_at	YGL056C	SDS23	4,3	0,00E+00	1
3647_f_at	YNL337W	---	4,3	0,00E+00	1
8187_at	YOR343C	---	4,3	0,00E+00	1
9631_at	YMR009W	ADI1	4,3	9,10E-12	1
7843_at	YPL092W	SSU1	4,3	0,00E+00	1
4400_at	YHR150W	PEX28	4,2	0,00E+00	1
7431_at	YBL065W	---	4,2	0,00E+00	1
4846_at	YGR146C	ECL1	4,2	0,00E+00	1
10687_at	YKL097C	---	4,2	0,00E+00	7
10500_at	YKR075C	---	4,2	0,00E+00	7
3629_f_at	YNR077C	---	4,2	0,00E+00	7
7782_at	YPL016W	SWI1	4,2	0,00E+00	7
10085_at	YLR237W	THI7	4,2	0,00E+00	8
4388_at	YHR139C	SPS100	4,1	0,00E+00	1
11141_at	YJL106W	IME2	4,1	0,00E+00	8
7958_at	YPL200W	CSM4	4,1	0,00E+00	1
8430_at	YOR138C	RUP1	4,1	0,00E+00	8
10776_at	YKL187C	---	4,0	0,00E+00	1
9696_at	YML058W	SML1	4,0	0,00E+00	7
4211_at	YIL099W	SGA1	4,0	0,00E+00	1
9424_at	YMR197C	VTI1	4,0	0,00E+00	1
7068_at	YBR295W	PCA1	4,0	0,00E+00	1
9885_at	YLR438W	CAR2	4,0	0,00E+00	1
9668_at	YML042W	CAT2	4,0	0,00E+00	1
10184_at	YLR154C	RNH203	4,0	0,00E+00	7
9065_at	YNL194C	---	4,0	0,00E+00	1
4124_at	YIL047C	SYG1	3,9	0,00E+00	7
10058_at	YLR256W	HAP1	3,9	0,00E+00	7
6839_at	YCR051W	---	3,9	0,00E+00	7
9034_at	YNL179C	---	3,9	0,00E+00	1
7107_at	YBR242W	---	3,9	0,00E+00	7
7313_at	YBR045C	GIP1	3,8	0,00E+00	1
5125_at	YGL117W	---	3,8	0,00E+00	8
4326_at	YHL030w-a	---	3,8	0,00E+00	8
7545_at	YPR193C	HPA2	3,8	0,00E+00	8
8551_at	YOL011W	PLB3	3,7	0,00E+00	1
9662_at	YML007W	YAP1	3,7	0,00E+00	1
4862_at	YGR161C	RTS3	3,7	0,00E+00	1
6449_at	YDR034W-B	---	3,7	0,00E+00	1
6527_at	YDL071C	---	3,7	0,00E+00	7
4978_at	YGR052W	FMP48	3,7	0,00E+00	1
7643_at	YPR111W	DBF20	3,7	0,00E+00	8
9639_at	YMR017W	SPO20	3,7	0,00E+00	1
7760_at	YPR008W	HAA1	3,7	0,00E+00	8
5314_at	YFL021c-a	---	3,7	0,00E+00	1
8688_at	YOL146W	PSF3	3,7	0,00E+00	7
6168_at	YDR294C	DPL1	3,6	0,00E+00	8
7890_at	YPL135W	ISU1	3,6	0,00E+00	1
5258_at	YGL255W	ZRT1	3,6	0,00E+00	8
6428_at	YDR059C	UBC5	3,6	0,00E+00	1

5400_at	YFL030W	AGX1	3,6	0,00E+00	1
8654_at	YOL089C	HAL9	3,5	0,00E+00	8
6472_at	YDR011W	SNQ2	3,5	0,00E+00	7
4756_at	YGR236C	SPG1	3,5	0,00E+00	1
10111_at	YLR219W	MSC3	3,5	0,00E+00	1
10026_at	YLR311C	---	3,5	0,00E+00	1
10614_at	YKL038W	RGT1	3,5	0,00E+00	1
10262_at	YLR053C	---	3,5	0,00E+00	1
5755_at	YEL035C	UTR5	3,5	0,00E+00	1
3975_at	NC_001224	COB	3,5	0,00E+00	1
7410_at	YBL042C	FUI1	3,5	0,00E+00	6
8206_at	YOR316C	COT1	3,4	0,00E+00	8
3972_s_at	NC_001224	BI4 /// COB	3,4	0,00E+00	1
5039_at	YGL023C	PIB2	3,4	0,00E+00	8
5973_at	YDR504C	SPG3	3,4	0,00E+00	1
9762_at	YML123C	PHO84	3,4	0,00E+00	8
6271_at	YDR214W	AHA1	3,4	0,00E+00	1
5422_s_at	YFL067W	---	3,4	0,00E+00	7
3853_f_at	YJR162C	---	3,3	0,00E+00	7
4759_at	YGR239C	PEX21	3,3	0,00E+00	8
6397_at	YDR073W	SNF11	3,3	0,00E+00	1
4464_at	YHR082C	KSP1	3,3	0,00E+00	1
8659_at	YOL084W	PHM7	3,3	0,00E+00	1
6273_at	YDR216W	ADR1	3,3	0,00E+00	1
10492_at	YKR067W	GPT2	3,3	0,00E+00	1
9124_at	YNL270C	ALP1	3,3	1,36E-10	1
6100_at	YDR359C	EAF1	3,3	0,00E+00	7
4546_at	YHL016C	DUR3	3,3	0,00E+00	1
5584_at	YER144C	UBP5	3,3	0,00E+00	1
8572_at	YOL035C	---	3,3	6,03E-11	1
8237_at	YOR303W	CPA1	3,3	0,00E+00	7
5137_at	YGL153W	PEX14	3,2	0,00E+00	1
9972_at	YLR348C	DIC1	3,2	0,00E+00	8
11266_f_at	YAL069W	---	3,2	1,68E-09	7
9066_at	YNL193W	---	3,2	0,00E+00	8
9063_at	YNL196C	SLZ1	3,2	0,00E+00	1
7291_at	YBR068C	BAP2	3,2	0,00E+00	1
10236_at	YLR117C	CLF1	3,2	0,00E+00	8
4246_at	YIL155C	GUT2	3,2	0,00E+00	1
4316_at	YHR202W	---	3,2	0,00E+00	1
6637_at	YDL138W	RGT2	3,2	0,00E+00	8
7593_at	YPR150W	---	3,1	0,00E+00	1
9615_at	YMR037C	MSN2	3,1	0,00E+00	7
7869_at	YPL111W	CAR1	3,1	0,00E+00	1
7732_at	YPR025C	CCL1	3,1	0,00E+00	1
7214_at	YBR167C	POP7	3,1	0,00E+00	8
9198_s_at	YNL333W	SNZ2 /// SNZ3	3,1	0,00E+00	1
7168_at	YBR212W	NGR1	3,1	0,00E+00	1
8490_at	YOR062C	---	3,1	0,00E+00	1
5961_at	YDR492W	IZH1	3,1	0,00E+00	8
5071_at	YGL080W	FMP37	3,1	0,00E+00	1
6209_at	YDR244W	PEX5	3,1	0,00E+00	1
10538_at	YKR021W	ALY1	3,1	0,00E+00	1
4542_at	YHL020C	OPI1	3,0	0,00E+00	8
7478_s_at	YBL109W	---	3,0	0,00E+00	1
10932_at	YJR094C	IME1	3,0	0,00E+00	1
6120_at	YDR334W	SWR1	3,0	5,92E-13	7
8960_at	YNL116W	DMA2	3,0	0,00E+00	8
10854_at	YJR152W	DAL5	3,0	0,00E+00	1
8019_s_at	YPL277C	---	3,0	0,00E+00	1
10620_at	YKL032C	IXR1	3,0	0,00E+00	1
9542_at	YMR100W	MUB1	3,0	0,00E+00	8
4160_at	YIL057C	---	3,0	0,00E+00	1
9158_at	YNL281W	HCH1	3,0	0,00E+00	7
9377_at	YMR238W	DFG5	3,0	0,00E+00	7
6528_at	YDL070W	BDF2	3,0	0,00E+00	1
4507_at	YHR033W	---	2,9	0,00E+00	8

6693_at	YDL215C	GDH2	2,9	0,00E+00	1
8165_at	YOR366W	---	2,9	0,00E+00	1
10476_at	YKR096W	---	2,9	0,00E+00	1
8924_at	YNL064C	YDJ1	2,9	0,00E+00	6
4705_at	YGR275W	RTT102	2,9	0,00E+00	7
10875_at	YJR127C	RSF2	2,8	0,00E+00	1
9976_at	YLR352W	---	2,8	5,60E-11	1
6195_at	YDR275W	BSC2	2,8	0,00E+00	8
6934_at	YCL041C	---	2,8	0,00E+00	1
11026_at	YJR004C	SAG1	2,8	0,00E+00	1
10126_at	YLR191W	PEX13	2,8	1,48E-08	8
5528_at	YER179W	DMC1	2,8	1,02E-08	8
8819_at	YNR060W	FRE4	2,8	1,74E-12	1
10388_at	YLL043W	FPS1	2,8	0,00E+00	7
3325_f_at	YER188c-a	---	2,8	1,17E-12	1
4194_at	YIL117C	PRM5	2,8	5,37E-08	7
5974_at	YDR505C	PSP1	2,8	0,00E+00	1
6803_at	YCR062W	---	2,8	0,00E+00	1
6401_at	YDR077W	SED1	2,8	0,00E+00	1
7956_at	YPL202C	AFT2	2,8	1,74E-12	1
9049_at	YNL164C	IBD2	2,8	0,00E+00	7
6190_at	YDR270W	CCC2	2,8	0,00E+00	6
3356_f_at	YDR543C	---	2,8	0,00E+00	7
8453_at	YOR116C	RPO31	2,8	1,33E-09	6
10704_at	YKL124W	SSH4	2,7	0,00E+00	1
10496_at	YKR071C	DRE2	2,7	0,00E+00	7
7739_at	YPR031W	NTO1	2,7	5,92E-13	8
8537_at	YOR019W	---	2,7	0,00E+00	1
11163_at	YJL132W	---	2,7	0,00E+00	1
6137_at	YDR351W	SBE2	2,7	6,84E-12	7
10480_at	YKR099W	BAS1	2,7	1,17E-12	6
8517_at	YOR044W	IRC23	2,7	0,00E+00	7
10210_at	YLR136C	TIS11	2,7	0,00E+00	1
5173_at	YGL162W	SUT1	2,7	0,00E+00	8
4761_at	YGR241C	YAP1802	2,7	0,00E+00	1
5096_at	YGL101W	---	2,7	3,57E-11	6
7339_at	YBR026C	ETR1	2,7	0,00E+00	1
9970_at	YLR346C	---	2,7	0,00E+00	1
6847_g_at	YCR061W	---	2,7	0,00E+00	1
7846_at	YPL089C	RLM1	2,7	5,71E-12	1
5074_at	YGL077C	HNM1	2,7	1,41E-11	6
4763_at	YGR243W	FMP43	2,7	0,00E+00	1
5748_at	YEL042W	GDA1	2,7	0,00E+00	6
9549_at	YMR107W	SPG4	2,7	0,00E+00	1
5101_at	YGL096W	TOS8	2,7	0,00E+00	1
10724_at	YKL149C	DBR1	2,6	0,00E+00	1
7270_i_at	YBR090C	---	2,6	0,00E+00	1
8888_at	YNL007C	SIS1	2,6	0,00E+00	1
8974_at	YNL103W	MET4	2,6	3,61E-08	1
9764_at	YML121W	GTR1	2,6	0,00E+00	7
9196_s_at	YNL335W	DDI2 /// DDI3	2,6	0,00E+00	1
8787_at	YNR032C-A	HUB1	2,6	0,00E+00	7
8895_at	YNL047C	SLM2	2,6	0,00E+00	8
6549_at	YDL049C	KNH1	2,6	0,00E+00	8
6647_at	YDL173W	PAR32	2,6	4,51E-11	8
5154_at	YGL134W	PCL10	2,6	1,74E-12	8
7184_at	YBR183W	YPC1	2,6	0,00E+00	1
4581_at	YHL027W	RIM101	2,6	0,00E+00	8
5685_at	YER030W	CHZ1	2,6	0,00E+00	7
10179_at	YLR149C	---	2,6	0,00E+00	1
10514_at	YKR042W	UTH1	2,6	0,00E+00	7
7919_at	YPL151C	PRP46	2,6	0,00E+00	1
9403_at	YMR221C	---	2,6	0,00E+00	6
6883_at	YCR005C	CIT2	2,6	0,00E+00	8
5409_at	YFL021W	GAT1	2,6	0,00E+00	7
5789_at	YEL046C	GLY1	2,6	2,64E-10	8
7774_at	YPL024W	RMI1	2,6	5,18E-10	8

9161_at	YNL278W	CAF120	2,6	0,00E+00	8
7631_at	YPR101W	SNT309	2,6	0,00E+00	1
8643_at	YOL100W	PKH2	2,6	1,02E-11	1
7403_at	YBL049W	MOH1	2,5	0,00E+00	1
11077_at	YJL035C	TAD2	2,5	0,00E+00	7
10792_at	YKL220C	FRE2	2,5	1,52E-11	1
5201_at	YGL179C	TOS3	2,5	0,00E+00	8
10910_at	YJR117W	STE24	2,5	0,00E+00	7
10748_at	YKL171W	---	2,5	0,00E+00	1
7974_at	YPL230W	USV1	2,5	1,17E-12	1
7196_at	YBR150C	TBS1	2,5	0,00E+00	8
9123_at	YNL271C	BNI1	2,5	5,56E-08	7
8200_at	YOR356W	---	2,5	0,00E+00	8
9347_at	YMR296C	LCB1	2,5	0,00E+00	6
7544_at	YPR192W	AQY1	2,5	6,36E-11	1
8144_g_at	YOR389W	---	2,5	0,00E+00	1
5420_s_at	YFL068W	---	2,5	1,25E-11	7
4391_at	YHR142W	CHS7	2,5	0,00E+00	7
3970_s_at	NC_001224	BI3 /// BI4 /// COB	2,5	6,17E-10	1
10315_at	YLR019W	PSR2	2,5	0,00E+00	7
10744_at	YKL175W	ZRT3	2,5	0,00E+00	8
11357_at	YAL053W	FLC2	2,5	7,97E-12	8
10945_at	YJR061W	---	2,5	0,00E+00	1
9288_s_at	YMR323W	ERR1 /// ERR2 /// ERR3	2,5	0,00E+00	1
4338_at	YHR178W	STB5	2,5	0,00E+00	8
9308_at	YMR302C	YME2	2,5	0,00E+00	1
8655_at	YOL088C	MPD2	2,5	5,92E-13	8
6576_at	YDL110C	TMA17	2,5	0,00E+00	1
6341_at	YDR150W	NUM1	2,5	0,00E+00	1
4440_at	YHR102W	KIC1	2,5	0,00E+00	7
9199_at	YNL331C	AAD14	2,5	0,00E+00	1
4396_at	YHR146W	CRP1	2,5	0,00E+00	1
5332_at	YFR033C	QCR6	2,5	0,00E+00	1
5701_at	YER002W	NOP16	2,5	0,00E+00	6
6233_at	YDR222W	---	2,5	4,58E-12	6
6821_at	YCR082W	AHC2	2,4	0,00E+00	1
6333_at	YDR142C	PEX7	2,4	0,00E+00	1
7733_at	YPR026W	ATH1	2,4	0,00E+00	1
10368_s_at	YLL020C	KNS1	2,4	0,00E+00	1
4140_at	YIL031W	ULP2	2,4	2,00E-09	1
8297_at	YOR231W	MKK1	2,4	0,00E+00	7
9992_at	YLR323C	CWC24	2,4	0,00E+00	8
5968_at	YDR499W	LCD1	2,4	4,02E-12	8
9502_at	YMR147W	---	2,4	0,00E+00	8
4976_at	YGR050C	---	2,4	0,00E+00	8
4385_at	YHR136C	SPL2	2,4	7,34E-11	1
7975_at	YPL229W	---	2,4	0,00E+00	1
8882_at	YNL013C	---	2,4	0,00E+00	1
4576_at	YHL032C	GUT1	2,4	0,00E+00	1
9554_at	YMR069W	NAT4	2,4	1,74E-12	7
5241_at	YGL228W	SHE10	2,4	0,00E+00	1
5778_at	YEL057C	---	2,4	0,00E+00	1
5182_at	YGL152C	---	2,4	4,11E-10	1
11017_at	YJL004C	SYS1	2,4	0,00E+00	8
9586_at	YMR056C	AAC1	2,4	0,00E+00	1
6161_at	YDR329C	PEX3	2,4	0,00E+00	1
7404_at	YBL048W	RRT1	2,4	0,00E+00	1
5737_at	YEL009C	GCN4	2,4	0,00E+00	7
10411_f_at	YLL065W	---	2,4	7,62E-08	7
8030_at	YPL265W	DIP5	2,4	0,00E+00	1
7982_at	YPL222W	FMP40	2,4	0,00E+00	1
11359_at	YAL049C	AIM2	2,4	0,00E+00	1
7122_at	YBR257W	POP4	2,4	1,17E-12	8
7623_at	YPR135W	CTF4	2,4	0,00E+00	8
10477_g_at	YKR096W	---	2,4	1,40E-03	1
10548_at	YKR031C	SPO14	2,4	0,00E+00	1
8889_at	YNL006W	LST8	2,4	0,00E+00	1

11386_at	YAL062W	GDH3	2,4	0,00E+00	1
10942_at	YJR103W	URA8	2,4	0,00E+00	1
8608_at	YOL044W	PEX15	2,4	0,00E+00	1
11134_at	YJL115W	ASF1	2,3	0,00E+00	7
8815_at	YNR056C	BIO5	2,3	3,47E-10	8
10377_at	YLL053C	---	2,3	0,00E+00	6
6862_at	YCR028C	FEN2	2,3	5,92E-13	8
4437_at	YHR100C	GEP4	2,3	7,12E-11	8
8143_at	YOR389W	---	2,3	6,57E-11	1
4872_at	YGR127W	---	2,3	0,00E+00	1
7268_at	YBR090C-A	NHP6B	2,3	0,00E+00	1
11308_s_at	YAR068W	---	2,3	3,48E-10	8
8973_at	YNL104C	LEU4	2,3	0,00E+00	8
8229_at	YOR297C	TIM18	2,3	1,74E-12	7
6544_at	YDL054C	MCH1	2,3	0,00E+00	1
9989_at	YLR320W	MMS22	2,3	4,12E-06	8
7398_at	YBL054W	TOD6	2,3	2,68E-06	6
7832_at	YPL057C	SUR1	2,3	5,92E-13	1
8941_at	YNL091W	NST1	2,3	0,00E+00	1
7318_at	YBR050C	REG2	2,3	0,00E+00	1
5810_s_at	YEL074W	---	2,3	2,31E-12	1
8364_at	YOR207C	RET1	2,3	0,00E+00	6
8186_at	YOR342C	---	2,3	0,00E+00	6
4754_at	YGR234W	YHB1	2,3	6,52E-11	6
6907_at	YCL025C	AGP1	2,3	0,00E+00	1
6649_at	YDL171C	GLT1	2,3	0,00E+00	8
10522_at	YKR050W	TRK2	2,3	1,88E-07	1
7789_at	YPL054W	LEE1	2,3	0,00E+00	1
3974_at	NC_001224	COB	2,3	2,05E-07	1
4750_at	YGR274C	TAF1	2,3	0,00E+00	6
6497_at	YDL010W	GRX6	2,3	0,00E+00	8
8365_at	YOR208W	PTP2	2,3	1,17E-12	1
8607_at	YOL045W	PSK2	2,3	0,00E+00	7
5995_at	YDR481C	PHO8	2,3	0,00E+00	1
8536_at	YOR018W	ROD1	2,3	2,36E-11	8
6565_at	YDL078C	MDH3	2,3	0,00E+00	1
10471_at	YKR092C	SRP40	2,3	9,66E-12	6
5120_at	YGL122C	NAB2	2,3	0,00E+00	1
7807_at	YPL036W	PMA2	2,3	5,92E-13	1
5560_at	YER167W	BCK2	2,3	3,89E-09	8
9067_at	YNL192W	CHS1	2,3	1,63E-11	7
9473_at	YMR161W	HLJ1	2,2	0,00E+00	7
10706_at	YKL123W	---	2,2	3,02E-11	1
5381_at	YFL007W	BLM10	2,2	0,00E+00	1
8935_at	YNL052W	COX5A	2,2	0,00E+00	1
6513_at	YDL039C	PRM7	2,2	7,97E-12	6
7426_at	YBL071C	---	2,2	6,95E-08	7
11149_at	YJL144W	---	2,2	2,41E-11	1
6024_at	YDR464W	SPP41	2,2	5,92E-13	8
5388_at	YFR001W	LOC1	2,2	6,04E-07	6
6227_at	YDR262W	---	2,2	0,00E+00	1
7983_at	YPL221W	FLC1	2,2	0,00E+00	8
10534_at	YKR017C	---	2,2	2,41E-04	1
9324_at	YMR316W	DIA1	2,2	1,04E-09	7
4204_at	YIL107C	PFK26	2,2	2,30E-11	1
5215_at	YGL210W	YPT32	2,2	2,88E-12	8
10703_at	YKL125W	RRN3	2,2	7,97E-12	6
7772_at	YPL026C	SKS1	2,2	7,01E-11	6
6729_at	YDL223C	HBT1	2,2	0,00E+00	1
9664_at	YML005W	TRM12	2,2	3,64E-10	8
9056_at	YNL203C	---	2,2	1,41E-11	1
8401_at	YOR154W	SLP1	2,2	5,32E-07	8
5382_at	YFL006W	BLM10	2,2	0,00E+00	1
8477_at	YOR095C	RKI1	2,2	5,92E-13	6
8459_at	YOR078W	BUD21	2,2	0,00E+00	6
5166_at	YGL169W	SUA5	2,2	4,85E-09	6
10678_at	YKL106W	AAT1	2,2	1,17E-12	6

10466_at	YKR087C	OMA1	2,2	0,00E+00	8
8228_at	YOR338W	---	2,2	1,91E-11	1
10117_at	YLR225C	---	2,2	6,91E-10	7
8179_at	YOR380W	RDR1	2,2	1,17E-12	1
10290_at	YLR038C	COX12	2,2	0,00E+00	1
9318_at	YMR310C	---	2,2	1,60E-09	6
5966_at	YDR497C	ITR1	2,2	0,00E+00	6
4091_at	YIR010W	DSN1	2,2	2,05E-08	6
6026_at	YDR420W	HKR1	2,2	5,92E-13	6
9538_at	YMR096W	SNZ1	2,2	0,00E+00	1
9754_at	YML091C	RPM2	2,2	9,83E-09	1
9778_at	YML109W	ZDS2	2,2	5,92E-13	8
9017_at	YNL151C	RPC31	2,2	2,74E-09	6
6399_at	YDR075W	PPH3	2,2	3,04E-06	6
8863_at	YNR015W	SMM1	2,2	8,53E-12	6
10862_s_at	YJR160C	MPH2 /// MPH3	2,1	4,04E-10	1
11118_at	YJL084C	ALY2	2,1	5,15E-12	1
6363_at	YDR128W	MTC5	2,1	0,00E+00	8
7290_at	YBR067C	TIP1	2,1	0,00E+00	1
6846_at	YCR061W	---	2,1	0,00E+00	1
7170_at	YBR168W	PEX32	2,1	0,00E+00	1
5703_at	YER004W	FMP52	2,1	0,00E+00	1
4255_at	YIL146C	ATG32	2,1	5,92E-13	1
5200_at	YGL180W	ATG1	2,1	8,10E-11	1
6595_at	YDL133W	---	2,1	0,00E+00	8
8617_at	YOL081W	IRA2	2,1	4,45E-11	1
4220_at	YIL136W	OM45	2,1	0,00E+00	1
10559_at	YKL005C	BYE1	2,1	3,34E-09	1
10765_at	YKL197C	PEX1	2,1	0,00E+00	1
11334_at	YAL032C	PRP45	2,1	2,25E-11	8
7094_at	YBR276C	PPS1	2,1	0,00E+00	8
10328_at	YLL015W	BPT1	2,1	1,02E-09	1
8435_at	YOR098C	NUP1	2,1	0,00E+00	7
6004_at	YDR443C	SSN2	2,1	9,47E-09	8
9552_at	YMR110C	HFD1	2,1	9,13E-11	1
8220_at	YOR330C	MIP1	2,1	5,15E-12	8
4079_at	YIL003W	CFD1	2,1	0,00E+00	8
9476_at	YMR164C	MSS11	2,1	1,12E-04	1
8218_at	YOR328W	PDR10	2,1	0,00E+00	1
6467_at	YDR006C	SOK1	2,1	6,98E-08	1
9028_at	YNL186W	UBP10	2,1	0,00E+00	6
4162_at	YIL055C	---	2,1	5,92E-13	1
8597_at	YOL054W	PSH1	2,1	0,00E+00	8
9433_at	YMR206W	---	2,1	0,00E+00	1
8373_at	YOR171C	LCB4	2,1	2,69E-06	6
5169_at	YGL166W	CUP2	2,1	0,00E+00	8
7833_at	YPL056C	---	2,1	5,92E-13	1
7319_at	YBR051W	---	2,1	6,99E-06	1
7296_at	YBR073W	RDH54	2,1	7,41E-12	6
8457_at	YOR076C	SKI7	2,1	0,00E+00	8
8872_at	YNL023C	FAP1	2,1	2,01E-08	6
9873_at	YLR426W	---	2,1	1,17E-12	8
10369_at	YLL019C	KNS1	2,1	0,00E+00	1
7730_at	YPR023C	EAF3	2,1	0,00E+00	1
4226_at	YIL130W	ASG1	2,1	5,44E-11	6
9575_at	YMR042W	ARG80	2,1	1,00E-10	8
8433_at	YOR141C	ARP8	2,1	0,00E+00	1
7749_at	YPL004C	LSP1	2,1	0,00E+00	1
10359_at	YLL027W	ISA1	2,1	0,00E+00	1
7750_at	YPL003W	ULA1	2,1	0,00E+00	1
4121_at	YIL006W	YIA6	2,1	5,15E-08	8
5658_at	YER047C	SAP1	2,1	0,00E+00	1
4742_at	YGR266W	---	2,1	1,69E-11	1
6517_at	YDL035C	GPR1	2,1	5,46E-08	8
7063_at	YBR290W	BSD2	2,1	5,47E-08	8
5049_at	YGL058W	RAD6	2,1	5,92E-13	7
8458_at	YOR077W	RTS2	2,0	4,10E-09	8

9109_at	YNL240C	NAR1	2,0	0,00E+00	1
8834_at	YNR031C	SSK2	2,0	1,09E-08	6
10746_at	YKL173W	SNU114	2,0	2,88E-12	8
4427_at	YHR091C	MSR1	2,0	4,02E-12	6
10884_at	YJR136C	TTI2	2,0	2,11E-08	8
4957_at	YGR030C	POP6	2,0	2,54E-07	8
5261_at	YGL252C	RTG2	2,0	2,88E-12	8
9116_at	YNL232W	CSL4	2,0	0,00E+00	6
5642_at	YER069W	ARG5,6	2,0	0,00E+00	6
7464_at	YBL078C	ATG8	2,0	0,00E+00	1
9287_s_at	YMR322C	HSP32 /// HSP33 /// SN	2,0	1,69E-11	1
11232_at	YJL194W	CDC6	2,0	2,34E-06	6
9595_at	YMR065W	KAR5	2,0	1,09E-09	1
7706_at	YPR042C	PUF2	2,0	0,00E+00	1
4770_at	YGR250C	---	2,0	1,53E-10	1
11101_at	YJL056C	ZAP1	2,0	2,57E-09	8
11083_at	YJL074C	SMC3	2,0	1,19E-09	8
10095_at	YLR247C	IRC20	2,0	1,72E-07	1
10542_at	YKR025W	RPC37	2,0	5,00E-11	6
5326_at	YFR029W	PTR3	2,0	3,17E-08	8
7386_at	YBL021C	HAP3	2,0	3,41E-08	6
8310_at	YOR244W	ESA1	2,0	0,00E+00	1
5770_at	YEL064C	AVT2	2,0	8,98E-07	8
9982_at	YLR357W	RSC2	2,0	1,28E-08	6
9902_at	YLR411W	CTR3	2,0	4,02E-12	1
10645_at	YKL052C	ASK1	2,0	1,75E-11	8
6282_at	YDR180W	SCC2	2,0	8,94E-07	6
9638_at	YMR016C	SOK2	2,0	0,00E+00	7
6196_at	YDR276C	PMP3	2,0	0,00E+00	7
11377_at	YAL034C	FUN19	2,0	5,92E-13	1
10465_at	YKR086W	PRP16	2,0	5,32E-07	6
4129_at	YIL042C	PKP1	2,0	5,92E-13	1
11065_at	YJL048C	UBX6	2,0	0,00E+00	1
10108_at	YLR216C	CPR6	2,0	0,00E+00	1
10688_at	YKL097W-A	CWP2	2,0	5,81E-07	1
4524_at	YHR006W	STP2	2,0	0,00E+00	8
4412_at	YHR119W	SET1	2,0	1,66E-04	8
6505_at	YDL046W	NPC2	2,0	0,00E+00	1
4579_at	YHL029C	OCA5	2,0	5,92E-13	6
4582_at	YHL026C	---	2,0	7,23E-08	7
11050_at	YJL018W	MPS3	2,0	5,33E-11	8
8883_at	YNL012W	SPO1	2,0	5,61E-10	1
10349_at	YLR007W	NSE1	2,0	3,93E-10	8
4065_at	YIR029W	DAL2	2,0	1,30E-11	1
